# Pathological response in resectable non–small cell lung cancer: a systematic literature review and meta-analysis

**DOI:** 10.1093/jncics/pkae021

**Published:** 2024-03-23

**Authors:** Nathalie A Waser, Melanie Quintana, Bernd Schweikert, Jamie E Chaft, Lindsay Berry, Ahmed Adam, Lien Vo, John R Penrod, Joseph Fiore, Donald A Berry, Sarah Goring

**Affiliations:** Insights, Evidence and Value, ICON plc, Burlington, ON, Canada; Berry Consultants LCC, Houston, TX, USA; Insights, Evidence and Value, ICON plc, Munich, Germany; Department of Medicine, Memorial Sloan Kettering Cancer Center, New York, NY, USA; Berry Consultants LCC, Houston, TX, USA; Insights, Evidence and Value, ICON plc, Burlington, ON, Canada; Health Economics and Outcomes Research, Bristol Myers Squibb, Lawrenceville, NJ, USA; Health Economics and Outcomes Research, Bristol Myers Squibb, Lawrenceville, NJ, USA; Health Economics and Outcomes Research, Bristol Myers Squibb, Lawrenceville, NJ, USA; Berry Consultants LCC, Houston, TX, USA; Insights, Evidence and Value, ICON plc, Burlington, ON, Canada

## Abstract

**Background:**

Surrogate endpoints for overall survival in patients with resectable non–small cell lung cancer receiving neoadjuvant therapy are needed to provide earlier treatment outcome indicators and accelerate drug approval. This study’s main objectives were to investigate the association among pathological complete response, major pathological response, event-free survival and overall survival and to determine whether treatment effects on pathological complete response and event-free survival correlate with treatment effects on overall survival.

**Methods:**

A comprehensive systematic literature review was conducted to identify neoadjuvant studies in resectable non–small cell lung cancer. Analysis at the patient level using frequentist and Bayesian random effects (hazard ratio [HR] for overall survival or event-free survival by pathological complete response or major pathological response status, yes vs no) and at the trial level using weighted least squares regressions (hazard ratio for overall survival or event-free survival vs pathological complete response, by treatment arm) were performed.

**Results:**

In both meta-analyses, pathological complete response yielded favorable overall survival compared with no pathological complete response (frequentist, 20 studies and 6530 patients: HR = 0.49, 95% confidence interval = 0.42 to 0.57; Bayesian, 19 studies and 5988 patients: HR = 0.48, 95% probability interval = 0.43 to 0.55) and similarly for major pathological response (frequentist, 12 studies and 1193 patients: HR = 0.36, 95% confidence interval = 0.29 to 0.44; Bayesian, 11 studies and 1018 patients: HR = 0.33, 95% probability interval = 0.26 to 0.42). Across subgroups, estimates consistently showed better overall survival or event-free survival in pathological complete response or major pathological response compared with no pathological complete response or no major pathological response. Trial-level analyses showed a moderate to strong correlation between event-free survival and overall survival hazard ratios (*R*^2^ = 0.7159) but did not show a correlation between treatment effects on pathological complete response and overall survival or event-free survival.

**Conclusion:**

There was a strong and consistent association between pathological response and survival and a moderate to strong correlation between event-free survival and overall survival following neoadjuvant therapy for patients with resectable non–small cell lung cancer.

Within the resectable non–small cell lung cancer (NSCLC) setting, clinical studies in which overall survival is the primary endpoint require many years of follow-up. Thus, there is growing interest in early endpoints such as pathological complete response, major pathologic response, and event-free survival, and the strength of their relationship with overall survival in patients receiving neoadjuvant treatment (1-3). Over the past several years, immunotherapies targeting the programmed cell death 1 protein have become additional options as neoadjuvant, adjuvant, or perioperative therapy for the treatment of NSCLC (4-9). In CheckMate 816, where pathological complete response is a primary endpoint, an exploratory analysis showed that pathological response could be an early indicator of event-free survival benefit in patients treated with immunotherapy as well as, in this case, nivolumab plus chemotherapy (10). In patients with resectable NSCLC (6-9,11), immunotherapies combined with chemotherapy before surgery followed by adjuvant immunotherapy showed significant improvements in pathological complete response and event-free survival relative to placebo with chemotherapy before surgery followed by placebo. Reliable early endpoints following neoadjuvant therapy would provide an early indicator of prognosis, potentially aid decision making in delivering adjuvant therapy, and allow expedited development of promising therapeutics. Pathological complete response appears to be a promising early endpoint; recent meta-analyses and a real-world study in patients treated with chemotherapy or chemoradiation therapy (CRT) have shown a 50% or more reduction in mortality and progression events for patients with pathological complete response compared with those without pathological complete response (12-14).

In breast cancer, pathological complete response has been used to successfully support an early regulatory approval of neoadjuvant treatment (2,3,15). Given the association observed between pathological complete response and event-free survival/overall survival at the patient level (despite the lack of an association at the trial level) and the long time needed to demonstrate overall survival benefit in a randomized controlled trial, the US Food and Drug Administration issued guidance (originally in 2014 and updated in 2020) that pathological complete response can be used as a surrogate endpoint in high-risk early-stage breast cancer to support accelerated approval (15).

According to the International Conference on Harmonization Guidelines on Statistical Principles in Clinical Trials, validation of surrogacy requires 1) biological plausibility of the relationship; 2) demonstration of the surrogate prognostic value for the clinical outcome (patient-level association), and 3) evidence from clinical trials that treatment effects on the surrogate correspond to effects on the clinical outcome (trial-level association). The International Conference on Harmonization further clarifies that the relationship between clinical and surrogate variables for 1 therapy does not necessarily apply to a therapy with a different mechanism of action. The International Association for the Study of Lung Cancer has ongoing multidisciplinary initiatives, including the collection of individual patient-level data, to tackle the issue of surrogacy in neoadjuvant resectable NSCLC aiming to establish pathological complete response and major pathological response as predictors of long-term clinical benefit (16,17).

In this paper, we extend the work previously done on NSCLC (ie, pathological response to event-free survival or overall survival) in several ways. We extended the synthesis of the literature-based evidence to investigate a larger number of relationships, including major pathological response to overall survival and major pathological response to event-free survival (at the patient level) and pathological complete response to overall survival, pathological complete response to event-free survival, and event-free survival to overall survival (at the trial level), and use additional methods to confirm the magnitude of the relationships using both frequentist and Bayesian models.

## Methods

###  

#### Systematic literature review

A search of MEDLINE, Embase, and Cochrane Central Register of Controlled Trials was run from database inception to March 11, 2019. International conferences were searched for 2 years before March 2019. Search strategies used controlled vocabulary (eg, Medical Subject Headings terms) and free-text terms. Eligibility criteria according to Population, Intervention, Comparison, Outcomes and Study were adult patients with resectable stage I to III NSCLC, with or without variations (population); neoadjuvant therapies (ie, chemotherapy, targeted therapy, or immunotherapy) with or without radiotherapy (RT) (intervention/comparator); pathological complete response or major pathological response and overall survival or event-free survival (outcomes); and randomized controlled trials, single-arm trials, and observational studies (study design). Event-free survival was defined as either disease-free survival, progression-free survival, or recurrence-free survival because these definitions were often used interchangeably. Two independent reviewers (A.A. and N.W.) reviewed records against these eligibility criteria, and adjudication was made by a third reviewer (S.G.) in cases of disagreement.

#### Objectives

The primary objectives were to 1) determine whether pathological response was associated with overall survival and event-free survival (ie, pathological complete response and major pathological response [patient-level analyses, frequentist and Bayesian analyses]), 2) model treatment effects on event-free survival and overall survival by estimating the expected improvement in event-free survival or overall survival for an improvement in the proportion of patients with tumors demonstrating a pathological complete response or major pathological response (patient-level analyses, Bayesian), 3) determine whether treatment effects on pathological complete response or major pathological response are correlated with treatment effects on overall survival or event-free survival (trial-level analyses), and 4) determine whether treatment effects on event-free survival are correlated with treatment effects on overall survival (trial-level analyses). The secondary objective was to identify study characteristics and endpoint definitions that could influence the relationship between endpoints.

#### Endpoint definitions and measures

Endpoint definitions, as described in the publications, were compared against established standards (18). According to those standards, pathological complete response was defined as 0% viable tumor cells by pathological assessment of the resected specimen (including primary tumor and lymph nodes), and major pathological response was defined as 10% or less viable tumor cells in the resected specimen (including primary tumor and lymph nodes). Disease-free survival, recurrence-free survival, and progression-free survival were most often defined as time to recurrence or death, except in 1 instance where second lung cancer was included as an event in addition to recurrence and death. There was variability, however, in what was considered time zero in the survival analyses, time zero being time of surgery, time of diagnosis, or starting time of neoadjuvant chemotherapy.

The hazard ratio (HR) for overall survival or event-free survival by pathological complete response or major pathological response status (ie, pathological complete response vs no pathological complete response or major pathological response vs no major pathological response), hazard ratio for overall survival or event-free survival by treatment arm (ie, treatment A vs treatment B), and pathological complete response or major pathological response rates were abstracted from the publications. Where both univariate and multivariate hazard ratios were published, the univariate value was used in the analysis or figures. When the hazard ratio was not available, it was derived from the reconstructed Kaplan-Meier curves by digitizing the published curves, extracting the number at risk and the number of events, and using the Guyot et al. approach to derive patient-level survival data (19). Reconstructed Kaplan-Meier curves were compared with the published curves to check the accuracy of reconstruction. The quality of the hazard ratios was categorized according to whether they were author reported (and thus based on raw data), estimated using derived patient-level survival data in which information about censoring was available, or estimated using derived patient-level survival data in which information about censoring was unavailable. The method by Altman and Bland et al. was used, which suggested the use of *P* values to calculate (log) SEs and 95% confidence intervals (CIs) when hazard ratios were reported without an associated confidence interval (20). No other imputations were performed.

#### Patient-level analyses

To quantify the association between pathological complete response or major pathological response and event-free survival or overall survival using patient-level summary statistics, a classical pairwise meta-analysis was first conducted within a frequentist framework. The meta-analysis incorporated all eligible studies that presented event-free survival or overall survival according to pathological complete response or major pathological response status, irrespective of treatment received. Relative effect estimates were captured as hazard ratios and 95% CIs or SEs of the log hazard ratio between patients’ groups: 1) those with tumors demonstrating a pathological complete response vs no pathological complete response and 2) those with tumors demonstrating a major pathological response vs no major pathological response. An overall summary hazard ratio estimate was produced, along with a 95% CI. A hazard ratio below 1.00 represented better event-free survival or overall survival associated with those patients whose tumors demonstrated a pathological complete response/major pathological response compared with no pathological complete response or no major pathological response. The random-effects framework was selected because of the assumption of heterogeneity across studies. Between-studies variance was determined using the DerSimonian-Laird method. *I*^2^ and Cochran *Q* heterogeneity statistics measured heterogeneity across studies, with *I*^2^ representing the proportion of dispersion that is expected to be real. As per Cochrane, *I*^2^ values can be grouped according to importance of heterogeneity and interpreted as follows: not important (0%-40%), moderate (30%-60%), substantial (50%-90%), and considerable (75%-100%) (21).

A similar patient-level meta-analysis of the association between pathological complete response or major pathological response and event-free survival or overall survival was implemented using a Bayesian hierarchical random-effects model. The Bayesian model was also used to predict future trial-level treatment effects on event-free survival or overall survival, given observed differences on pathological complete response or major pathological response. To implement the Bayesian model, a log hazard ratio was modeled for pathological complete response vs no pathological complete response (or major pathological response vs no major pathological response) with a noninformative, normally distributed prior with mean of 0 and SD of 10. Unlike the frequentist meta-analysis performed on study-level effects, the Bayesian model was fit to time-to-event outcomes (event-free survival or overall survival) using derived patient-level data reconstructed from published Kaplan-Meier curves. Details of the hierarchical Bayesian model are provided in Supplementary Methods (available online). Posterior distributions were estimated for each parameter using a Metropolis-Hastings within a Gibbs sampling algorithm custom coded in R statistical software (R Foundation for Statistical Computing, Vienna, Austria). Results included the fitted survival curves (posterior median and 95% probability interval) for pathological complete response vs no pathological complete response (and major pathological response vs no major pathological response), by study and overall, and the hazard ratio for pathological complete response vs no pathological complete response (and major pathological response vs no major pathological response) (posterior median and 95% probability interval).

Finally, to address the secondary objective, the meta-analyses were run in subgroups, including study design, geography, population characteristics, type of neoadjuvant treatment received (chemotherapy vs CRT), use of adjuvant therapy (yes vs no), pathological complete response definition (ie, ypT0N0 vs ypT0 vs 0% viable tumor cells), major pathological response definition (≤10% viable tumor cells vs other definitions), quality of hazard ratio summary measure, and time zero on Kaplan-Meier curves.

#### Trial-level analyses

Using the randomized controlled trial evidence only, weighted-linear regressions were performed to determine the correlation between the treatment effect on overall survival or event-free survival, measured as log hazard ratios for treatment A vs treatment B, and the treatment effect on pathological complete response, measured as risk differences for treatment A vs treatment B (22). An alternative correlation between the log of overall survival/event-free survival and the log of the odds ratio was also investigated. Because some odds ratios were undefined wherever pathological complete response was 0% (eg, in the surgery-alone arm) and the odds ratio range was limited compared with the risk difference range, the risk difference approach was selected as the preferred approach.

Similarly, the correlation between the treatment effect on overall survival and the treatment effect on event-free survival was assessed by fitting weighted linear regressions of overall survival summary measures (ie, hazard ratio) on event-free survival summary measures (ie, hazard ratio) using the same approach. Adjustments were necessary to calculate odds ratios for pathological complete response for randomized controlled trials that reported a pathological complete response of 0% in 1 arm (ie, pathological complete response of 0.5% instead of 0% in the surgery-alone arm). There was an insufficient number of studies to perform regressions of overall survival/event-free survival on major pathological response (2 randomized controlled trials).

The weights in the regression were defined by the randomized controlled trial sample size. The results were summarized by estimates of the slope, including SE and the *P* value testing the null hypothesis of the slope parameter being zero. For each survival vs pathological complete response relationship, the corresponding *R*^2^ and adjusted *R*^2^ statistics, along with the Pearson correlation coefficients, which in this case equal the square root of the (unadjusted) *R*^2^ statistic (each with a 95% CI), were calculated for each combination of pathological complete response and survival. For the overall survival vs event-free survival relationship, *R*^2^ and adjusted *R*^2^ statistics were used. According to the Institute for Quality and Efficiency in Health Care, when based on a highly reliable evidence base, a surrogate has a proven lack of validity if the upper limit of the r value’s 95% CI is 0.7 or less (*R*^2^ ≤ 0.49). The institute considers that a surrogate has proven validity only when the lower limit of the r value’s 95% CI is 0.85 or higher (*R*^2^ ≥ 0.72) (23).

## Results

### Patient-level analyses—association between overall survival or event-free survival and pathological complete response or major pathological response

A total of 31 studies, including 26 observational cohorts studies, 3 single-arm trials, and 2 randomized controlled trials, were included in the patient-level analyses (Table 1). The eligible population was either potentially resectable NSCLC, where patients did not necessarily proceed with surgical resection (ie, some patients had an exploratory thoracotomy), or resected NSCLC. Neoadjuvant treatment included chemotherapy with or without RT followed by surgery, with all neoadjuvant therapies consisting of platinum-based (ie, cisplatin or carboplatin) chemotherapy. In more recent studies, platinum agents were typically combined with docetaxel, paclitaxel, gemcitabine, vinorelbine, etoposide, or pemetrexed; in studies initiated in the 1990s cisplatin was often provided in combination with mitomycin, vindesine, ifosfamide, or fluorouracil.

**Table 1.** Study and patient characteristics in 31 studies included in patient-level analyses (frequentist and Bayesian)

Author (y)	**Included in at least 1 frequentist analysis** ^a^	**Included in at least 1 Bayesian analysis** ^a^	Study type	Study period	Country	Stage	No.	Eligible population	Neoadjuvant systemic therapy
**Neoadjuvant chemotherapy**									
Brandt (2019) (26)	Yes	Yes	Cohort	2000-2015	United States	IB-IIIA	184	Resected	Platinum doublet^b^
Cascone (2018) (43)	Yes	Yes	Other trial	2007-2009	United States	I-III	47	Potentially resectable	Cisplatin-docetaxel
Mouillet (2012) (44)	Yes	Yes	Randomized controlled trials	1991-2006	France	IB-IIB	492	Potentially resectable	Platinum-based chemotherapy^c^
Pataer (2012) (45)	Yes	Yes	Cohort	2001-2006	United States	I-IV	358	Resected	Platinum doublet^b^
Qu (2019) (46)	Yes	Yes	Cohort	2006-2014	United States	II-III	272	Resected	Platinum doublet^b^
Remark (2016) (35)	Yes	Yes	Cohort	2008-2012	France	III	161	Resected	Platinum doublet^b^
Spaggiari (2016) (31)	Yes	No	Cohort	1998-2013	Italy	IIIA	141	Potentially resectable	Cisplatin-based chemotherapy
Stefani (2010) (33)	Yes	No	Cohort	2001-2007	France	IIIA-IIIB	175	Resected	Platinum doublet^b^
**Neoadjuvant chemoradiation therapy**									
Appel (2017) (47)	Yes	Yes	Cohort	2012-2016	Israel	IIB-IIIB	52	Resected	Platinum doublet^b^
Coroller (2017) (34)	Yes	Yes	Cohort	2003-2013	United States	II-III	85	Resected	Not available
Fischer (2008) (36)	Yes	Yes	Cohort	1999-2007	Canada	IIB-IIIB	44	Resected	Cisplatin-etoposide
Haque (2019) (25)	Yes	Yes	Cohort	2004–2015	United States	III	1750	Resected	Not available
Isobe (2012) (28)	Yes	Yes	Other trial	2001-2010	Japan	III	30	Potentially resectable	Carboplatin-docetaxel
Kim (2016) (48)	Yes	Yes	Cohort	1997-2013	Korea	IIIA-IIIB	574	Resected	Platinum doublet^b^
Kim (2011) (49)	Yes	Yes	Cohort	1989-2008	United States	IB-IIIB	233	Resected	Platinum-based chemotherapy^d^
Lee (2012) (29)	Yes	No	Cohort	2004-2009	Korea	IIIA	205	Resected	Cisplatin-etoposide
Lee (2014) (30)	Yes	No	Cohort	1997-2011	Korea	IIIA	355	Potentially resectable	Platinum doublet^b^
Pöttgen (2015) (50)	Yes	Yes	Cohort	2000-2012	Germany	III	157	Potentially resectable	Cisplatin-based chemotherapy
Shintani (2012) (51)	Yes	Yes	Cohort	1995-2008	Japan	IIIA-IIIB	52	Potentially resectable	Cisplatin-based chemotherapy^e^
Shiraishi (2014) (27)	No	Yes	Cohort	1993-2011	Japan	IIB-IIIB	26	Resected	Platinum-based chemotherapy^f^
Tanaka (2018) (52)	Yes	Yes	Other trial	2011-2013	Japan	IIIA	40	Potentially resectable	Carboplatin-docetaxel
van der Meij (2011) (32)	Yes	No	Cohort	2003-2009	Netherlands	III	51	Resected	Cisplatin-based chemotherapy
Yamaguchi (2013) (53)	Yes	Yes	Cohort	2005-2011	Japan	IIIA/B	42	Potentially resectable	Cisplatin-tegafur, gimeracil, oteracil
Yokomise (2007) (24)	Yes	Yes	Randomized controlled trial	2000-2006	Japan	IIIA-IIIB	41	Potentially resectable	Carboplatin-taxane
**Neoadjuvant chemotherapy/chemoradiation therapy**									
Couñago (2019) (54)	Yes	Yes	Cohort	2005-2014	Spain	IIIA	118	Potentially resectable	Platinum doublet^b^
Kayawake (2019) (55)	Yes	Yes	Cohort	2005-2015	Japan	II-III	145	Resected	Carboplatin-based chemotherapy
Krantz (2018) (56)	Yes	Yes	Cohort	2006-2012	United States	IIIA	1945	Resected	Not available
Li (2009) (57)	Yes	Yes	Cohort	1998-2004	China	IIIA	91	Resected	Cisplatin-based chemotherapy
Martin (2002) (58)	Yes	Yes	Cohort	1993-1999	United States	IA-IV	446	Potentially resectable	Platinum-based chemotherapy^g^
Sawabata (2003) (59)	Yes	Yes	Cohort	1988-1999	Japan	IIIA-IIIB	131	Resected	Not available
Schreiner (2019) (60)	Yes	Yes	Cohort	2008-2017	Germany	IIIA/B	55	Resected	Cisplatin- etoposide

a The study inclusion status by analysis is detailed in Supplementary Table 1 (available online).

b Platinum doublet consisted of carboplatin or cisplatin in combination with 1 other agent, including vinorelbine, gemcitabine, etoposide, paclitaxel, docetaxel, or pemetrexed. Cisplatin-based chemotherapy and carboplatin-based chemotherapy could be doublets or triplets. Regimens that are no longer used as part of standard-of-care chemotherapy are detailed for each study.

c Mouillet et al. (2012) pooled results from the randomized controlled trials of Depierre et al. (2002) and Westeel et al. (2013) (61, 62). In trial 1 of Depierre et al (2002), neoadjuvant therapy consisted of cisplatin, ifosfamide, and mitomycin combination, while in trial 2 of Westeel et al. (2013), it was platinum doublets (ie, cisplatin-gemcitabine or carboplatin-paclitaxel) (44).

d In Kim et al. (2011), therapy was a mix of cisplatin, fluorouracil, and etoposide; carboplatin, paclitaxel, and etoposide; and carboplatin and paclitaxel (49).

e In Shintani et al. (2012), therapy consisted of cisplatin in triple combination with vindesine and mitomycin or in double combination with vindesine or vinorelbine (51).

f In Shiraishi et al. (2014), therapy consisted of a mix of cisplatin alone; cisplatin in combination with docetaxel, vindesine, or fluorouracil; or carboplatin combined with docetaxel (27).

g In Martin et al. (2002), treatment consisted of cisplatin in triple combination with vinblastine and mitomycin, carboplatin-paclitaxel, or other (58).

#### Overall survival by pathological complete response status (pathological complete response vs no pathological complete response)

**Figure 1.**
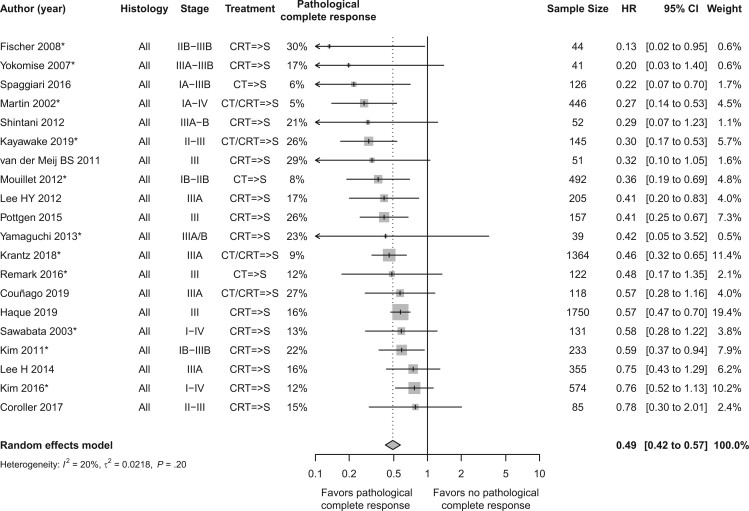
Frequentist analysis—overall survival by pathological complete response status (pathological complete response vs no pathological complete response). The authors of 2 studies reported both univariate and multivariate hazard ratio values (25,51). For both studies, the unadjusted hazard ratio value was plotted. The hazard ratio by multivariate analysis was 0.575 in Haque (2019) (thus similar to the plotted value of 0.57) (25) and 0.595 in the small study of Shintani (2012) (51). The hazard ratios in 3 studies were not plotted because they were 0, and the 95% confidence interval upper and lower bounds were also 0 (26-28). An asterisk means that the hazard ratio was reconstructed from Kaplan-Meier curves. => means followed by. CI = confidence interval; CRT = chemoradiation therapy; CT = chemotherapy; HR = hazard ratio; S = surgery.

For the frequentist approach, the hazard ratio for overall survival by pathological complete response status (pathological complete response vs no pathological complete response) across 20 studies (N = 6530) is presented in Figure 1. The list of studies included in the analysis is provided in Supplementary Table 1 (available online). The complete pathologic response ranged from 5% to 30%, and the number of patients with a pathological complete response ranged from 7 in Yokomise et al. (24) to 280 in Haque et al. (25). The overall hazard ratio for overall survival was 0.49 (95% CI = 0.42 to 0.57), indicating that the risk of death among patients with tumors demonstrating a pathological complete response was about half of that among patients whose tumors did not. The direction of the association was consistent across all studies, with hazard ratio point estimates ranging from 0.13 to 0.78. Ten of the 95% CIs were below 1.0. *I*^2^ was 20%, indicating that 80% of this observed variation may have been the result of sampling error rather than true heterogeneity and thus was categorized as unimportant according to Cochrane. The funnel plot for overall survival by pathological complete response status showed some asymmetry, indicating a possible publication bias (Supplementary Figure 1, available online).

**Figure 2.**
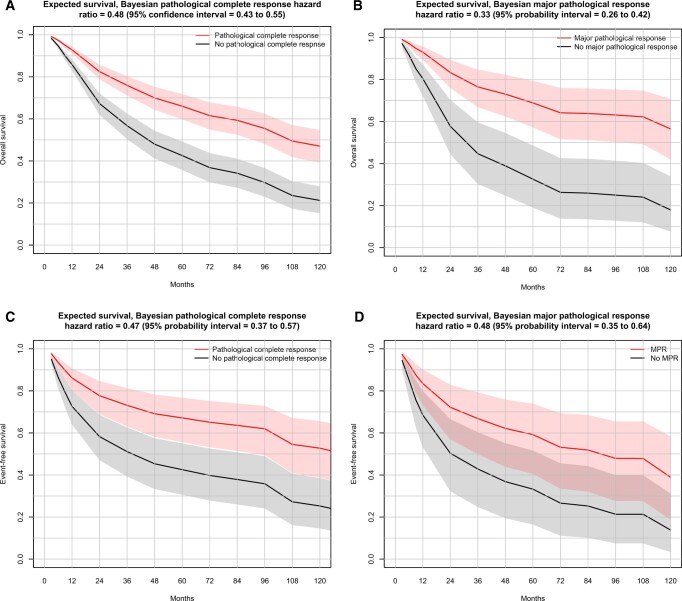
**A**) Bayesian analysis—expected overall survival, by pathological complete response status (median and 95% probability interval). These are the treatment effect predictions of a future randomized controlled trial based on results of Bayesian analysis of overall survival vs pathological complete response, assuming a 10% pathological complete response rate in the control arm. Solid lines represent posterior medians. Shaded regions represent 95% probability intervals. **B**) Bayesian analysis—expected overall survival, by major pathological response status (median and 95% probability intervals). These are the treatment effect predictions of a future randomized controlled trials based on results of Bayesian analysis of overall survival vs major pathological response, assuming a 10% major pathological response rate in the control arm. Solid lines represent posterior medians. Shaded regions represent 95% probability intervals. **C**) Bayesian analysis—expected event-free survival, by pathological complete response status (median and 95% probability interval). Treatment effect predictions of a future randomized controlled trial are based on results of Bayesian analysis of event-free survival vs pathological complete response and assume a 10% pathological complete response rate in the control arm. Solid lines represent posterior medians. Shaded regions represent 95% probability intervals. **D**) Bayesian analysis—expected event-free survival, by major pathological response status (median and 95% probability interval). Treatment effect predictions of a future randomized controlled trial are based on results of Bayesian analysis of event-free survival vs major pathological response and assume a 10% major pathological response rate in the control arm. Solid lines represent posterior medians. Shaded regions represent 95% probability intervals. MPR = major pathologic response.

Within the Bayesian analysis of overall survival by pathological complete response status, 19 studies were included (N = 5988). Three studies excluded from the frequentist analysis (26-28) were included in the analysis; however, 4 studies were excluded from the Bayesian analysis due to absence of published Kaplan-Meier curves (29-32). The estimated hazard ratio for overall survival by pathological complete response status is presented in Figure 2, A. The mean hazard ratio for overall survival by pathological complete response status (pathological complete response vs no pathological complete response) was 0.48 (95% PI: 0.43 to 0.55) with the Bayesian approach.

**Figure 3.**
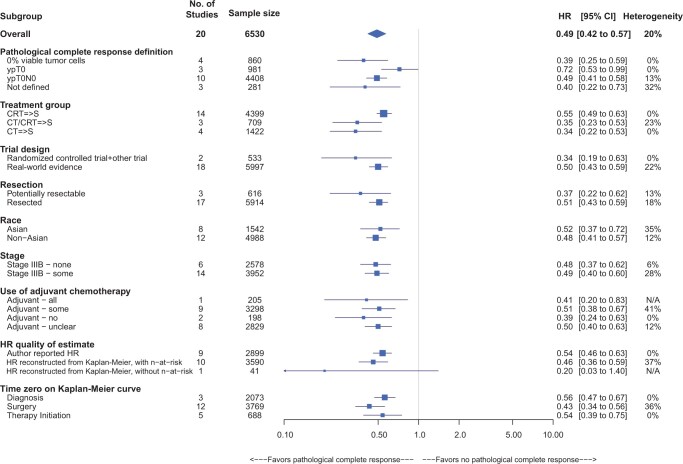
Frequentist analysis—subgroup analyses for overall survival, by pathological complete response status (pathological complete response vs no pathological complete response). The size of the square represents the study’s weight in the meta-analysis. The diamond represents the overall estimate. The taxane subgroup is not shown because only 1 study informed the taxane yes stratum. In the treatment subgroup, the number of studies does not add up to 20 but to 21 because Krantz et al. (2018) reported separately on subgroups CT=>S and CRT=>S (56). Thus the same study is counted in the 2 strata CT=>S and CRT=>S. => means followed by. CI = confidence interval; CRT = chemoradiation therapy; CT = chemotherapy; HR = hazard ratio; N/A = not applicable; S = surgery.

Subgroup analyses within the frequentist approach are presented in Figure 3. All meta-analyzed estimates within subgroups had hazard ratios below 1.00, and all hazard ratios in subgroups with sample sizes of more than 1 study had upper 95% CIs below 1.00. The only subgroup estimate with an upper 95% CI above 1.00 was based on low-quality hazard ratio reconstruction (due to nonreporting of n-at-risk) based on 1 small randomized controlled trial (N = 41) by Yokomise et al. (2007), which had a strong effect size (ie, HR = 0.20) yet was associated with considerable uncertainty (24).

The 95% CIs overlapped across all subgroups; there were no statistically significant differences across subgroup-specific hazard ratios. The association of pathological complete response with overall survival trended more strongly in certain subgroups, however, such as 1) the subgroup involving patients treated with neoadjuvant chemotherapy alone (HR = 0.34, 95% CI = 0.22 to 0.53) compared with the subgroup involving neoadjuvant CRT (HR = 0.55, 95% CI = 0.49 to 0.63); 2) patients with potentially resectable tumors (HR = 0.37, 95% CI = 0.22 to 0.62) relative to patients with resected tumors (HR = 0.51, 95% CI = 0.43 to 0.59); 3) patients who did not receive adjuvant therapy (HR = 0.39, 95% CI = 0.24 to 0.63) compared with those patients who received some adjuvant therapy (HR = 0.51, 95% CI = 0.38 to 0.67); and 4) studies where pathological complete response was defined as ypT0N0 (HR = 0.49, 95% CI = 0.41 to 0.58) compared with those studies where pathological complete response was defined as ypT0 (HR = 0.72, 95% CI = 0.53 to 0.99).

#### Overall survival by major pathological response status (major pathological response vs no-major pathological response)

**Figure 4.**
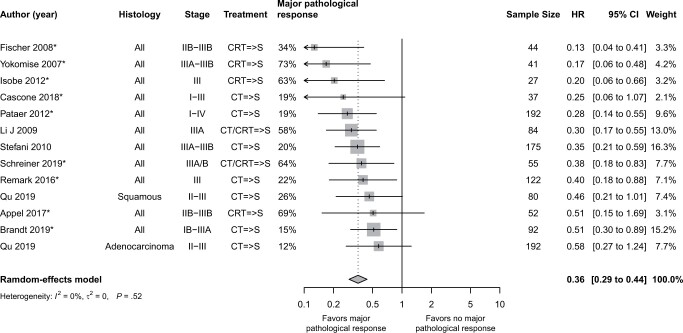
Frequentist analysis—hazard ratio for overall survival, by major pathological response status (major pathological response vs no major pathological response). Qu (2019) performed both multivariate and univariate analysis (46). For adenocarcinoma, the univariate analysis was not significant; thus, only a univariate hazard ratio was available, and it is the value plotted here (46). For the squamous histology, in addition to the univariate value, which is plotted here, the hazard ratio by multivariate analysis was 0.565 (46). In Li (2009), only the multivariate hazard ratio was reported, and that is the value plotted here (57). An asterisk means that the hazard ratio was reconstructed from Kaplan-Meier curves. => means followed by. CI = confidence interval; CRT = chemoradiation therapy; CT = chemotherapy; HR = hazard ratio; S = surgery.

The hazard ratio for overall survival by major pathological response status across 12 studies (N = 1193) is presented in Figure 4 for the frequentist approach. Overall, the hazard ratio for overall survival was 0.36 (95% CI = 0.29 to 0.44), the direction of association was consistent across studies, and heterogeneity in the real effect size was considered low (*I*^2^ = 0%). Within the Bayesian analysis, 11 studies were included (N = 1018) in the analysis of hazard ratios for overall survival by major pathological response status (Figure 2, B). One study could not be included because of the absence of published Kaplan-Meier curves (33). The mean overall hazard ratio for overall survival by major pathological response status was 0.33 (95% probability interval = 0.26 to 0.42) for the Bayesian analysis. Subgroup analyses consistently were in the direction of a better overall survival in major pathological response vs no major pathological response, and all 95% CIs were under 1.00, with the exception of the use of the adjuvant chemotherapy subgroup, where 1 stratum involved 1 small trial for which the hazard ratio was below 1.00 but was associated with considerable uncertainty (Supplementary Figure 2, available online).

#### Event-free survival by pathological complete response status (pathological complete response vs no pathological complete response)

The hazard ratio for event-free survival by pathological complete response status in 11 studies (N = 2156) is presented in Supplementary Figure 3 (available online) for the frequentist approach. The overall hazard ratio for event-free survival was 0.49 (95% CI = 0.41 to 0.60), with a consistent direction of association across studies and no heterogeneity. Within the Bayesian analysis, 8 studies were included (N = 1665) in the analysis of event-free survival by pathological complete response status (Figure 2, C). Three studies were excluded because of an absence of published Kaplan-Meier curves (30,32,34). The mean overall hazard ratio for event-free survival was 0.47 (95% probability interval = 0.37 to 0.57). The subgroup estimates yielded similar results within the frequentist approach (Supplementary Figure 4, available online). As with overall survival vs pathological complete response, the association between event-free survival and pathological complete response trended more strongly in the neoadjuvant chemotherapy subgroup than in the neoadjuvant CRT subgroup (ie, HR = 0.40, 95% CI = 0.22 to 0.73, compared with HR = 0.56, 95% CI = 0.44 to 0.72, respectively).

#### Event-free survival by major pathological response status (major pathological response vs no major pathological response)

Within the frequentist analysis, the hazard ratios for event-free survival by major pathological response status in 6 studies (N = 770) are presented in Supplementary Figure 5 (available online). The overall hazard ratio for event-free survival was 0.52 (95% CI = 0.42 to 0.66) and no heterogeneity. Within the Bayesian approach, 6 studies were included (N = 770) in the analysis of event-free survival by major pathological response status (Figure 2, D). The mean overall hazard ratio for event-free survival by major pathological response status (major pathological response vs no major pathological response) was 0.48 (95% probability interval = 0.35 to 0.64). Subgroup analyses show estimates consistently in the direction of better event-free survival in major pathological response vs no major pathological response, despite a lower number of studies (Supplementary Figure 6, available online).

#### Frequentist vs Bayesian results

A summary of the frequentist and Bayesian results is presented in Table 2. Overall, results were consistent across the 2 approaches. Numerically, the Bayesian estimates tended to be somewhat lower than those generated using a frequentist approach, possibly due to a difference in methods (eg, piecewise fitting of exponential survival curves or random-effects models) or data used in each approach (summary statistics or derived patient-level data). Subgroup analyses for the Bayesian meta-analysis compared with the frequentist meta-analysis, shown in Supplementary Table 2 (available online), were broadly similar.

**Table 2.** Frequentist and Bayesian meta-analyses summary results of associations between pathological complete response or major pathological response and overall survival or event-free survival

Association	Analysis	HR (95% CI or probability interval)	Lowest-highest HRs on forest plots	No. of patients	No. of studies
Overall survival, by pathological complete response status (pathological complete response vs no pathological complete response)	Frequentist	0.49 (0.42 to 0.57)	0.13-0.78	6530	20
	Bayesian	0.48 (0.43 to 0.55)	—^a^	5988	19
Event-free survival, by pathological complete response status (pathological complete response vs no pathological complete response)	Frequentist	0.49 (0.41 to 0.60)	0.26-0.71	2156	11
	Bayesian	0.47 (0.37 to 0.57)	—^a^	1665	8
Overall survival, by major pathological response status (major pathological response vs no major pathological response)	Frequentist	0.36 (0.29 to 0.44)	0.13-0.58	1193	12
	Bayesian	0.33 (0.26 to 0.42)	—^a^	1018	11
Event-free survival, by major pathological response status (major pathological response vs no major pathological response)	Frequentist	0.52 (0.42 to 0.66)	0.43-0.60	770	6
	Bayesian	0.48 (0.35 to 0.64)	—^a^	770	6

a Not available. CI = confidence interval, HR = hazard ratio.

### Bayesian predictions of treatment effects on overall survival based on treatment effects on pathological complete response

Within the Bayesian approach, the predicted hazard ratio and 95% probability interval (shaded region) for overall survival by treatment arm given a range of incremental benefits in pathological complete response rates between treatment arms is provided in Supplementary Figure 7 (available online). According to the predicted estimates from this model, the pathological complete response rate in the experimental arm must show an improvement of at least 30% to 35% compared with the control arm to achieve a hazard ratio for overall survival of 0.80 or less. Supplementary Table 2 (available online) shows the treatment difference in pathological complete response rates needed between 2 arms in a hypothetical randomized controlled trial to achieve a mean predicted hazard ratio for overall survival between treatment arms of 0.80 or less. Within the subgroups that have the strongest association (smallest hazard ratio for pathological complete response status) between pathological complete response and overall survival (ie, neoadjuvant chemotherapy, 0% viable tumor cells, and no adjuvant chemotherapy), the smallest predicted difference in pathological complete response between treatment arms of a hypothetical randomized clinical trial that is needed is 20% to 25% to achieve a mean hazard ratio for overall survival between treatment arms of 0.80 or less, using the historical chemotherapy-based data identified in this literature review.

### Trial-level analyses—correlation between treatment effect on pathological complete response and treatment effect on overall survival/event-free survival

**Figure 5.**
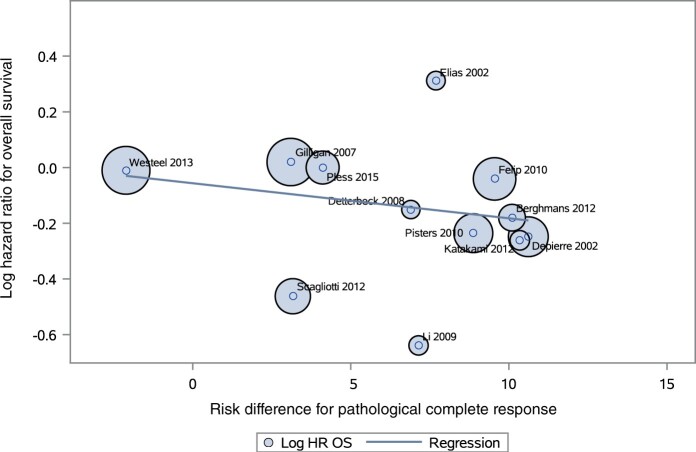
Trial-level analysis—hazard ratio for overall survival vs risk difference for pathological complete response. The line is the linear regression; a circle represents the weight of each trial. HR = hazard ratio; OS = overall survival.

Twelve of 18 randomized controlled trials were included in the regression analysis of hazard ratio for overall survival by treatment arm vs difference in pathological complete response by treatment arm (Supplementary Table 1, available online). There were insufficient major pathological response data to perform any correlation analysis. Details of the therapies received in the 18 randomized controlled trials are presented in Supplementary Table 3 (available online). Treatment arms included neoadjuvant chemotherapy with or without RT or surgery alone. Some randomized controlled trials included adjuvant chemotherapy, RT, or both in 1 treatment arm. Across the 12 randomized controlled trials, the pathological complete response was relatively low, ranging from 0% to 14%, and the difference in pathological complete response between arms was low (ie, 0%-10%). The log hazard ratio for overall survival vs the risk difference for pathological complete response rates is presented in Figure 5. The unadjusted trial-level coefficient of determination (*R*^2^) was 0.103 (95% CI = ‒0.174 to 0.379), and the Pearson coefficient r_Pearson_ was ‒0.322 (95% CI = ‒0.750 to 0.322). The odds ratio model is presented in Supplementary Figure 8 (available online). The odds ratio model results similarly showed an absence of correlation between pathological complete response and overall survival (*R*^2^ = 0.045, 95% CI = ‒0.149 to 0.239; Pearson coefficient r_Pearson_ = 0.211, 95% CI = ‒0.695 to 0.420).

Six of 18 randomized controlled trials were included in the regression analysis of hazard ratios for event-free survival by treatment arm vs pathological complete response (Supplementary Table 1, available online). The log hazard ratio for event-free survival vs log risk difference for pathological complete response is presented in Supplementary Figure 9 (available online). The trial-level coefficient of determination (*R*^2^) was 0.246 (95% CI = ‒0.222 to 0.714), and the Pearson coefficient r_Pearson_ was ‒0.496 (95% CI = ‒0.926 to 0.563). The odds ratio model is presented in Supplementary Figure 10 (available online). Results from the odds ratio model showed the lack of correlation (*R*^2^ = 0.319, 95% CI = ‒0.162 to 0.803; Pearson coefficient r_Pearson_ = ‒0.565, 95% CI = ‒0.937 to 0.499).

#### Trial-level analysis—correlation between treatment effect on overall survival and treatment effect on event-free survival

A total of 12 of 18 randomized controlled trials were included in the hazard ratios for overall survival vs hazard ratios for event-free survival regression (Supplementary Table 1, available online). Supplementary Figure 11 (available online) shows the linear regression and summary data. The trial-level *R*^2^ was 0.7159, showing a moderate to strong association between the relative treatment effect on overall survival and the relative treatment effect on event-free survival.

## Discussion

After performing frequentist and Bayesian meta-analyses on a comprehensive evidence base in resectable NSCLC treated with neoadjuvant chemotherapy or CRT, results showed that patients with tumors that demonstrated a pathological complete response had approximately half the mortality risk and half the risk of an event-free survival event compared with patients with tumors without a pathological complete response. This finding is consistent with recent meta-analyses that were based on random-effects and fixed-effects models and a recent real-world study using individual patient-level data specifically from community practices in the United States (12-14). A strong association between pathological complete response and event-free survival was also found in an exploratory analysis of CheckMate 816, the first immunotherapy phase III clinical trial conducted in patients treated with neoadjuvant nivolumab plus chemotherapy vs chemotherapy (HR = 0.13, 95% CI = 0.05 to 0.37) (5). Furthermore, the associations between major pathological response and overall survival and major pathological response and event-free survival were of similar magnitude and statistically significant, which indicates that tumor pathological complete response or major pathological response after neoadjuvant treatment was associated with longer overall survival and event-free survival.

The benefit was seen across all individual studies and in all subgroups where estimates were consistently in the direction of better overall survival in those patients with tumors that demonstrated a pathological complete response compared with those that did not. Interestingly, the association between pathological complete response and overall survival was slightly stronger in the 4 studies (N = 1422) in which patients were treated with neoadjuvant chemotherapy only (without RT) compared with the overall analysis (frequentist: HR for chemotherapy = 0.34, 95% CI = 0.22 to 0.53 vs HR overall = 0.49, 95% CI = 0.42 to 057; Bayesian: HR for chemotherapy = 0.32, 95% probability interval = 0.20 to 0.52 vs HR overall = 0.48, 95% probability interval = 0.43 to 0.55). Although the differences across subgroups were not statistically significant, there is a clinical rationale supporting this finding such that the local disease control achieved with neoadjuvant RT may have increased pathological complete response without substantially affecting distant recurrence and overall survival in this setting. Results from the adjuvant treatment subgroup analyses were inconclusive. The number of studies and patients were small in many of the subgroups. Also, in some of the studies, it is unclear whether adjuvant therapy was included; in others, although it was specified that some patients received adjuvant therapy, results are presented for the overall population only. More research is needed to determine the role of adjuvant therapy following neoadjuvant therapy.

Interestingly, the association between overall survival and major pathological response was numerically stronger than that between overall survival and pathological complete response (frequentist: 0.36 vs 0.49; Bayesian: 0.33 vs 0.48), although the analysis of major pathological response to overall survival association was based on a smaller number of studies than pathological complete response to overall survival. Because the population with 1% to 10% viable tumor cells is part of the no pathological complete response group, it might explain these results and suggest that major pathological response could be a stronger predictor of overall survival. The difference in estimates may also be due to heterogeneity or publication bias, however, rather than a true trend. In a subset of 5 studies that reported overall survival both by pathological complete response and major pathological response status, the hazard ratios were not consistently aligned with our findings [1 study reported a stronger effect by major pathological response (35), 2 reported a stronger effect by pathological complete response (26,28), and 2 reported a similar effect (24,36)].

Results from the Bayesian meta-analyses were used to predict treatment effects in survival based on treatment effects in response within the context of hypothetical randomized trials. Given the association found in this study, absolute differences of 30% to 35% in pathological complete response or major pathological response between 2 hypothetical treatment arms (or 20% to 25% if estimates from the neoadjuvant chemotherapy group are used, instead) would be required to achieve a clinically meaningful hazard ratio for survival of at least 0.80 between the 2 treatment arms. These predictions are likely conservative estimates because they assume that there is no additional treatment benefit beyond the increase in pathological complete response rates in the experimental group.

The trial-level analysis did not show any meaningful correlation between a treatment effect on pathological complete response and an improvement in overall survival or event-free survival. The lack of trial-level correlation is not surprising given both the low pathological complete response rates seen with neoadjuvant chemotherapy or CRT (ie, 0%-14%) and the small range in differences in pathological complete response between treatment groups observed in these randomized controlled trials (pathological complete response difference ranged from ‒2% to 10.3%). These trial-level findings in NSCLC were similar to previously published results in breast cancer, where the trial-level analysis failed to show a correlation (coefficient of determination was weak, and in Cortazar et al. the slope was in a direction that showed worse hazard ratios for overall survival or event-free survival, with increasing improvements in pathological complete response rates) (22,37). Although the association between pathological complete response and overall survival at the patient level is independent of treatment received (pathological complete response and non–pathological complete response groups are established, regardless of treatment), the trial-level analysis is likely highly dependent on the treatments being compared in this analysis. In the NSCLC analyses, the evidence was entirely based on chemotherapy (with or without RT), and differences in pathological complete response rates were small. In breast cancer, differences in pathological complete response between treatment arms were also small (1%-11%), except in the NeOAdjuvant Herceptin (NOAH) trial of trastuzumab (20%), an agent with a novel mechanism of action (38). The better trial-level results in NSCLC compared with breast cancer (ie, direction of slope) were possibly the result of the greater heterogeneity in tumor subtypes in breast cancer. The interaction between tumor subtypes and their respective treatment response may have complicated the trial-level analysis more in breast cancer than in NSCLC. Overall, the trial-level analysis presented here in NSCLC shares the same limitations as those mentioned in breast cancer. In addition, in NSCLC, there were fewer trials, and many trials were small in size, which may add to heterogeneity. Overall, caution should be exercised when interpreting and generalizing the trial-level analysis, and the correlation should be reexamined in coming years, when results from additional randomized controlled trials comparing differing mechanisms of action are available (eg, immunotherapy plus chemotherapy vs chemotherapy).

When a trial-level analysis was conducted to investigate the relative treatment effect on overall survival and event-free survival, a moderate to strong association was found. The association was also reported to be strong by Ostoros et al. in their primary analysis of randomized control trial evidence (39). A strong association between event-free survival and overall survival has also been previously reported with adjuvant chemotherapy in resected NSCLC (40). As many of the studies in this event-free survival or overall survival analysis compared different chemotherapy regimens (ie, chemotherapy vs chemotherapy), several hazard ratios clustered around 1. The correlation could be further explored when results are available from the many ongoing neoadjuvant trials evaluating new classes of therapeutics (programmed cell death 1 protein or programmed cell death 1 ligand 1, targeted therapies) vs chemotherapy, potentially yielding a wider range of event-free survival and overall survival results, although analysis of correlations should be restricted to particular drug classes because the relationship between endpoints may differ by mechanism of action (41).

Indeed, all analyses presented here were based on chemotherapy and CRT neoadjuvant treatments, which have shown only modest survival benefit vs no treatment in this setting (approximately 5% overall survival absolute risk reduction at 5 years) (42). However, immunotherapies have been shown to exhibit substantially different mechanism of action and durability of response compared with chemotherapy in metastatic NSCLC. Hence, it is possible that when using immunotherapy in an add-on approach, overall survival and event-free survival may be improved not only through the quantity of pathological complete responses and major pathological responses relative to chemotherapy but also through the quality and durability of the responses. Furthermore, it is possible that patients classified as having no pathological complete response or no major pathological response (such as those with some reduction of viable tumor but >10%) when treated with immunotherapy plus chemotherapy may derive additional benefit compared with those on chemotherapy alone. In CheckMate 816, event-free survival was longer in patients with pathological complete response than in no pathological complete response in both the immunotherapy plus chemotherapy and the chemotherapy arms (5). In patients without a pathological complete response, event-free survival was improved in the immunotherapy plus chemotherapy arm compared with the chemotherapy arm (median event-free survival = 26.6 months vs 18.4 months; HR for event-free survival = 0.84, 95% CI = 0.61 to 1.17; respectively).

A limitation of the current study was the reliance on published evidence and the lack of true patient-level data. Contrary to breast cancer, the individual patient data were not available from any of the studies, regardless of their design. This limitation is important one that would need to be addressed in future projects. Data were also limited because of the paucity of randomized controlled trials in the neoadjuvant setting, and many were conducted more than 20 years ago; thus, some treatment comparisons may not be relevant anymore. Another limitation is that the patient-level approach can only show associations, but there cannot be any inference of cause and effect. Indeed, if some patient characteristics were associated with pathological complete response, those same characteristics might be associated with improved survival.

This study investigated the magnitude of the association between early endpoints of pathological complete response, major pathological response, and event-free survival with overall survival and showed the existence of a strong and consistent patient-level association in resectable NSCLC treated with chemotherapy or CRT. At the trial level, there was a moderate to strong correlation between overall survival and event-free survival, but there appeared to be no correlation between overall survival or event-free survival and pathological complete response; the difference in the trial-level findings to those from patient-level analysis, especially regarding pathological complete response, may be due to the limited number of trials. Pathologic response is a promising endpoint for predicting survival, and further research is warranted to perform individual-patient data analyses and broaden the evidence-base to include new data from immunotherapy studies in the resectable NSCLC setting as well as those expected to report results in the near future.

## Supplementary Material

pkae021_Supplementary_Data

## Data Availability

Data used in this manuscript are available upon request from the corresponding author.
