# Supplementary material for: Pathological response in resectable non–small cell lung cancer: a systematic literature review and meta-analysis
Source: JNCI Cancer Spectr. 2024 Mar 23;8(3):pkae021. doi: 10.1093/jncics/pkae021 (PMC11101053; doi:10.1093/jncics/pkae021)
Supplement: pkae021_Supplementary_Data [file pkae021_supplementary_data.pdf]

Supplementary Methods

Patient-level Bayesian model for prediction of treatment effects

The Bayesian hierarchical random effect model synthesizes all available data and explicitly models heterogeneity between studies. The heterogeneity between studies within the Bayesian framework was estimated from the data provided. This allowed greater “borrowing” across studies if the results were similar and less “borrowing” if the results were different.

Time-to-event outcomes for every patient were modelled using a piecewise exponential distribution with segments every 3 months for the first year and yearly thereafter. The baseline hazard for each segment was estimated with a non-informative, normally distributed prior distribution. The model included study-specific random effects that allowed the hazard rate to vary proportionally across study, and the variance of the random-effects distribution was estimated based on the observed data and a weakly informative inverse gamma prior distribution. As such, the estimated study heterogeneity depended on the empirical evidence, with greater “borrowing” across studies if the results were similar.

Within the Bayesian hierarchical random effects analysis, time-to-event outcomes (EFS and OS) for N total patients across all studies were modeled as piecewise exponential. The total segments for analysis, T, were defined as quarter-year segments for the first year and yearly segments thereafter. For segment t, $t=1,\dots,T$, and patient i, $i=1,\dots,N$, we denote the hazard rate η_{ti}. These hazard rates are patient-specific and are functions of the study the patient was enrolled to, $s(i)$ for $s = 1, \dots, S$, and the pCR status of the patient, $p(i) = 0$ if no pCR and $p(i) = 1$ if pCR:

$$\eta_{t,i} = \lambda_t \exp\left(\alpha_{s(i)} + \theta_{p(i)}\right), t = 1 \dots T, i = 1 \dots N.$$

The α_s are study-specific random effects for studies $s = 1 \dots S$. They allow the hazard rate to vary from study to study but within a hierarchical model:

$\alpha_s \sim Normal(0, \sigma_\alpha^2), s = 1 \dots S;$

$1/\sigma_\alpha^2 \sim Gamma(2,1).$

The variance parameter, σ_α^2, is regarded to be unknown with a weak, essentially non-informative inverse gamma prior distribution. As such, the estimated study heterogeneity depended on the empirical evidence, with greater "borrowing" across studies if the results were similar. A reference study *s* = 1 is defined arbitrarily to have $\alpha_1 = 0$.

Assuming $\theta_0 = 0$ for no-pCR. Therefore, θ_1 is the log HR for pCR versus no-pCR. The log-HR for pCR was modeled assuming a non-informative normal prior distribution:

$$\theta_1 \sim N(0,10^2).$$

Finally, parameter λ_t was the baseline hazard rate for each segment *t* for study $s = 1$. The segment-specific baseline hazard rates, λ_t, was modeled assuming independent and identical non-informative prior distributions

$$\lambda_t \sim Gamma(0.01, 1), t = 1 \cdots T.$$

Additionally, HR for pCR/MPR estimated from the Bayesian analysis were used to translate potential treatment differences in pCR/MPR in a future trial to expected treatment effects on survival endpoints. This analysis assumed that the same relationship between pCR/MPR and OS/EFS estimated in this Bayesian meta-analysis would occur in a future trial investigating a new therapy. Using simulation, OS/EFS hazard ratios were predicted based on an assumed 10% pCR/MPR rate and all expected differences in pCR/MPR rates for the treatment arm between 0% and 90% by increments of 5%. This analysis was performed separately for each survival endpoint (EFS/OS) and both pathological response endpoints (pCR/MPR). This prediction is a conservative estimate of the expected survival benefit because it assumes that all benefit on OS/EFS is driven by an increase in pCR/MPR in the experimental group with no additional OS/EFS benefit of the experimental treatment on the non-pCR/non-MPR subjects. For the prediction model an HR of 0.80 was assumed to be clinically significant.

The posterior median and 95% probability interval of simulated survival hazard ratios were reported as the predicted survival hazard ratio for each incremental difference in pCR rates.

This was presented in a plot that shows all improvements in pCR rates in 5% increments and the predicted survival HR (median and shaded 95% probability interval). As such, the magnitude of improvement in pCR/MPR rates that would produce a clinical benefit in each survival outcome was assessed. The width of the probability interval (shaded region) for the predicted survival hazard ratio was a function of the trial sample size and duration. Results for a trial with 240 total participants followed for a maximum of 6 years were presented.

Supplementary Table 1: Studies (N=50) Inclusion Status by Analysis

Author (year)	Frequentist analysis				Bayesian analysis				Trial-level analysis		
	OS/pCR	OS/MPR	EFS/pCR	EFS/MPR	OS/pCR	OS/MPR	EFS/pCR	EFS/MPR	OS - pCR	EFS - pCR	OS vs EFS
Appel (2017)[47]	No	Yes	No	No	No	Yes	No	No	No	No	No
Berghmans (2012)[63]	No	No	No	No	No	No	No	No	Yes	No	No
Brandt (2019)[26][a]	No	Yes	Yes	Yes	Yes	Yes	Yes	Yes	No	No	No
Cascone (2018)[43]	No	Yes	No	Yes	No	Yes	No	Yes	No	No	No
Chen (2013)[64]	No	No	No	No	No	No	No	No	No	No	Yes
Coroller (2017)[34]	Yes	No	Yes	No	Yes	No	No	No	No	No	No
Couñago (2019)[54]	Yes	No	Yes	No	Yes	No	Yes	No	No	No	No
Dautzenberg (1990)[65]	No	No	No	No	No	No	No	No	No	No	Yes
Depierre (2002)[61]	No	No	No	No	No	No	No	No	Yes	No	No
Detterbeck (2008)[66]	No	No	No	No	No	No	No	No	Yes	No	No
Elias (2002)[67]	No	No	No	No	No	No	No	No	Yes	No	No
Felip (2010)[68]	No	No	No	No	No	No	No	No	Yes	Yes	Yes
Fischer (2008)[36]	Yes	Yes	No	No	Yes	Yes	No	No	No	No	No
Gilligan (2007)[69]	No	No	No	No	No	No	No	No	Yes	No	Yes
Girard (2010)[70]	No	No	No	No	No	No	No	No	No	No	No
Gottfried (2008)[71]	No	No	No	No	No	No	No	No	No	No	Yes
Haque (2019)[25]	Yes	No	No	No	Yes	No	No	No	No	No	No
Isobe (2012)[28][a]	No	Yes	No	No	Yes	Yes	Yes	No	No	No	No
Katakami (2012)[72]	No	No	No	No	No	No	No	No	Yes	Yes	Yes
Kayawake (2019)[55]	Yes	No	Yes	No	Yes	No	Yes	No	No	No	No
Kim (2016)[48]	Yes	No	Yes	No	Yes	No	Yes	No	No	No	No
Kim (2011)[49]	Yes	No	No	No	Yes	No	No	No	No	No	No
Krantz (2018)[56]	Yes	No	No	No	Yes	No	No	No	No	No	No
Lee (2012)[29][b]	Yes	No	No	No	No	No	No	No	No	No	No
Lee (2014)[30][b]	Yes	No	Yes	No	No	No	No	No	No	No	No
Li (2009)[57]	No	Yes	No	No	No	Yes	No	No	No	No	No
Li (2009)[73]	No	No	No	No	No	No	No	No	Yes	No	No
Martin (2002)[58]	Yes	No	No	No	Yes	No	No	No	No	No	No
Mouillet (2012)[44][c]	Yes	No	Yes	No	Yes	No	Yes	No	No	No	No
Pataer (2012)[45]	No	Yes	No	Yes	No	Yes	No	Yes	No	No	No
Pisters (2010)[74]	No	No	No	No	No	No	No	No	Yes	Yes	Yes
Pless (2015)[75]	No	No	No	No	No	No	No	No	Yes	Yes	Yes
Pöttgen (2015)[50]	Yes	No	No	No	Yes	No	No	No	No	No	No
Qu (2019)[46]	No	Yes	No	Yes	No	Yes	No	Yes	No	No	No

Remark (2016)[35]	Yes	Yes	Yes	Yes	Yes	Yes	Yes	Yes	No	No	No
Roth (1994)[76]	No	No	No	No	No	No	No	No	No	No	No
Rosell (1994)[77]	No	No	No	No	No	No	No	No	No	No	Yes
Sawabata (2003)[59]	Yes	No	No	No	Yes	No	No	No	No	No	No
Scagliotti (2012)[78]	No	No	No	No	No	No	No	No	Yes	Yes	Yes
Schreiner (2019)[60]	No	Yes	No	Yes	No	Yes	No	Yes	No	No	No
Shintani (2012)[51]	Yes	No	No	No	Yes	No	No	No	No	No	No
Shiraishi (2014)[27][a]	No	No	No	No	Yes	No	No	No	No	No	No
Spaggiari (2016)[31][b]	Yes	No	No	No	No	No	No	No	No	No	No
Stefani (2010)[33][b]	No	Yes	No	No	No	No	No	No	No	No	No
Tanaka (2018)[52]	No	No	Yes	No	No	No	Yes	No	No	No	No
Thomas (2008)[79]	No	No	No	No	No	No	No	No	No	No	Yes
van der Meij (2011)[32]	Yes	No	Yes	No	No	No	No	No	No	No	No
Yamaguchi (2013)[53]	Yes	No	Yes	No	Yes	No	Yes	No	No	No	No
Yokomise (2007)[24]	Yes	Yes	No	No	Yes	Yes	No	No	No	No	No
Westeel (2013)[62]	No	No	No	No	No	No	No	No	Yes	Yes	Yes
**Total number of studies in each analysis**	**20**	**12**	**11**	**6**	**19**	**11**	**8**	**6**	**12**	**6**	**12**

Note: Apart from Mouillet et al. (2012) and Yokomise et al. (2007), none of the RCTs reported survival endpoints by pCR status (pCR vs no-pCR) or MPR status (MPR vs no-MPR). Thus only two RCTs were included in the frequentist and Bayesian analyses. In contrast, the authors of most RCTs (and none of the other studies) reported a study-level measure of OS or EFS by treatment arm and % of patients with pCR by treatment arm. Thus, trial-level analyses were conducted based on the RCT evidence only. None of the RCTs reported % of patients with MPR by treatment arm; thus no trial-level analyses could be performed to investigate the association between survival endpoints and MPR.

[a] Kaplan-Meier curves show no event in the pCR group (HR = 0) and thus the study could not be included in the frequentist analysis due to lack of distinct lower and upper 95% CI estimates. However, the study was included in Bayesian analysis given presence of Kaplan-Meier curves allowing reconstruction of survival patient-level data.

[b] Authors reported HR for OS (or EFS) by pCR (or MPR) status but did not report the Kaplan-Meier curves for these associations. Thus the study could be included in the frequentist analysis based on the published HR. However, the study could not included in the Bayesian analysis due to our inability to reconstruct survival patient-level data.

[c] Mouillet et al. (2012) study published OS and EFS data by pCR status for overall population derived from the two combined RCTs by Depierre et al. (2002) and Westeel (2013). No reporting of endpoint by pCR status was available in the original publications of Depierre et al. (2002) and Westeel et al. (2013), thus frequentist and Bayesian analysis were conducted

based on the Mouillet study. However, OS (or EFS) and pCR were reported by treatment arm in the original publications of Depierre et al. (2002) and Westeel et al. (2013), thus the original publications were used for the trial-level analysis.

Abbreviations: EFS: Event-free survival; MPR: Major pathologic response; OS: Overall survival; pCR: Pathologic complete response; RCT: Randomized controlled trial.

Supplementary Table 2: Summary of Frequentist vs Bayesian Subgroup Analyses – HR for OS by pCR Status (pCR vs no-pCR)

Subgroup type	Subgroup detail	Frequentist Analysis (20 studies, N=6,530)				Bayesian Analysis (19 studies, N=5,988)				
		HR	95% CI	Sample Size	N of studies	HR	95% PI	Study Tx Diff. pCR for OS HR ≤0.80	Sample Size	N of studies
Overall population	Overall populatio	0.49	[0.42,0.57]	6530	20	0.48	[0.43,0.55]	0.30	5988	19
Tx group	CRT=>S	0.55	[0.49,0.63]	4399	14	0.52	[0.46,0.60]	0.35	3710	12[a]
	CT/CRT=>S	0.35	[0.23,0.53]	709	3	0.39	[0.27,0.54]	0.25	840	4
	CT=>S	0.34	[0.22,0.53]	1422	4	0.32	[0.20,0.52]	0.25	1438	4[a]
Presence of adjuvant CT	No adjuvant	0.39	[0.24,0.63]	198	2	0.35	[0.21,0.54]	0.25	340	3
	Some adjuvant	0.51	[0.38,0.67]	3298	9	0.50	[0.41,0.61]	0.35	2844	8
	All adjuvant	0.41	[0.20,0.83]	205	1	-[b]	-[b]	-[b]	-[b]	-[b]
	Unclear adjuvant	0.50	[0.40,0.63]	2829	8	0.50	[0.42,0.59]	0.35	2804	8
pCR definition	0% viable tumor cells	0.39	[0.25,0.59]	860	4	0.28	[0.16,0.44]	0.20	708	5
	ypT0	0.72	[0.53,0.99]	981	3	0.70	[0.47,0.97]	0.60	626	2
	ypT0N0	0.49	[0.41,0.58]	4408	10	0.49	[0.42,0.57]	0.35	4424	10
	Not defined	0.40	[0.22,0.73]	281	3	0.47	[0.30,0.69]	0.35	230	2
Time zero on KMC	Diagnosis	0.56	[0.47,0.67]	2073	3	0.57	[0.46,0.69]	0.45	1868	2
	Tx Initiation	0.54	[0.39,0.75]	688	5	0.45	[0.30,0.63]	0.30	502	6
	Surgery	0.43	[0.34,0.56]	3769	12	0.45	[0.37,0.54]	0.35	3618	11

[a]Number of studies does not add up to 19 in this subgroup analysis because Krantz et al. (2018) reported separately on subgroups CT=>S and CRT=>S. Thus this study is informing the 2 subgroups CT=>S and CRT=>S.

[b]Not available

Differences in pCR rates have been rounded to the nearest 0% or 5%

Legend: =>: followed by

Abbreviations: CI: Confidence interval for frequentist analysis, Probability interval for Bayesian analysis; CRT: Chemoradiotherapy; CT: Chemotherapy; HR: Hazard ratio; KMC: Kaplan-Meier curve; OS: Overall survival; pCR: Pathological complete response; RCT: Randomized controlled trial; S: Surgery; Tx: Treatment; ypT0: absence of disease in the primary tumor (T) at surgery; ypT0N0: absence of disease in primary

Supplementary Table 3: Summary of 18 RCTs (N=4,375) Reporting Relative or Absolute Treatment Effects on OS and EFS and used in trial-level analyses

Author Year	Name of RCT	Treatment Regimens		
		Tx Arm 1	Tx Arm 2	Tx Arm 3
Neoadjuvant platinum (carboplatin or cisplatin) doublets with or without RT				
Detterbeck (2008)[66]	GINEST 1&2	CIS-GEM=>S	GEM-TAX=>S	CARB-GEM=>S
Elias (2002)[67]	-[a]	CIS-ETO=>S=>CIS-ETO=> RT	RT=>S=>RT	-[b]
Felip (2010)[68]	NATCH	CARB-TAX=>S	S=>CARB-TAX	S
Girard (2010)[70]	IFCT 0101	CIS-GEM=>S	CIS-VNB+RT=>S	CARB-TAX+RT=>S
Katakami (2012)[72]	WJTOG9903	CARB-TXT+RT=>S	CARB-TXT=>S	-[b]
Li (2009)[73]	-[a]	CIS-VNB=>S	S	-[b]
Pisters (2010)[74]	SWOG S9900	CARB-TAX=>S	S	-[b]
Pless (2015)[75]	SAKK 16/00	CIS-TXT=>RT=>S	CIS-TXT=>S	-[b]
Scagliotti (2012)[78]	CHEST	CIS-GEM=>S	S	-[b]
Thomas (2008)[79]	GLCCG 01/95	CIS-ETO=>CARB-VDE+RT=>S=>RT	CIS-ETO=>S=>RT	-[b]
Westeel (2013)[62]	IFCT 0002	PD[d]=>S=>PD[d]	PD[d]=>S	-[b]
Neoadjuvant cisplatin triplets				
Chen (2013)[64]	-[a]	CIS-MIT-VDE=>S	S	-[b]
Dautzenberg (1990)[65]	-[a]	CIS-CYCLO-VDE=>S=>CIS-CYCLO-VDE	S	-[b]
Depierre (2002)[61]	-[a]	CIS-MIT-IFO=>S	S	-[b]
Gilligan (2007)[69]	MRC LU22	CT[c]=>S	S	-[b]
Gottfried (2008)[71]	-[a]	CIS-IFO-VNB=>S=>CIS-IFO-VNB	CIS-IFO-VNB=>S	-[b]
Roth (1994)[76]	-[a]	CIS-CYCLO-ETO=>S=>CIS-CYCLO-ETO	S	-[b]
Rosell (1994)[77]	-[a]	CIS-MIT-IFO=>S	S	-[b]

[a]No trial name provided

[b]Not applicable

[c]Neoadjuvant CT included a mix of old triple cisplatin-based chemotherapies (ie, CIS-MIT-VBA and CIS-MIT-IFO), and current CTs (ie, CIS-VNB, CARB-TAX, CIS-GEM or CARB-TXT)

[d]PD includes either CIS-GEM or CARB-TAX

Legend: +RT: concurrent RT; =>RT: Sequential RT; =>: followed by.

Abbreviations: CARB: Carboplatin; CIS: Cisplatin; CT: Chemotherapy; CYCLO: Cyclophosphamide; ETO: Etoposide; GEM: Gemcitabine; IFO: Ifosfamide; MIT: Mitomycin; PD: Platinum doublet; RCT: Randomized controlled trial; RT: Radiotherapy; S: Surgery; TAX: Paclitaxel; Tx: Treatment; TXT: Docetaxel; VBA: Vinblastine; VDE: Vindesine; VNB: Vinorelbine.

Supplementary Figure 1A: Funnel Plots of HR for OS by pCR Status (pCR vs no-pCR)

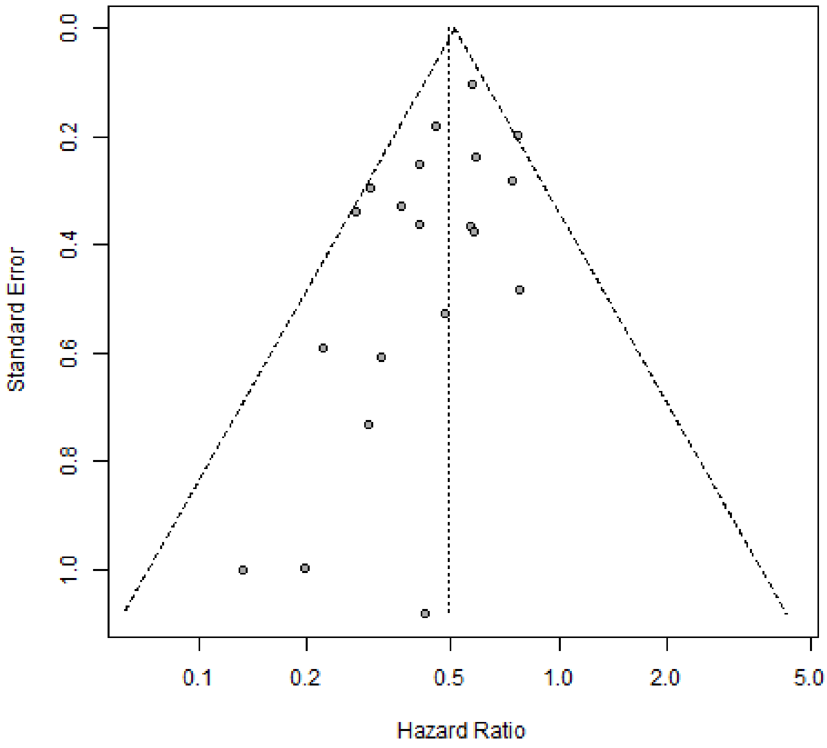

Supplementary Figure 1B: Funnel Plots of HR for EFS by pCR Status (pCR vs no-pCR)

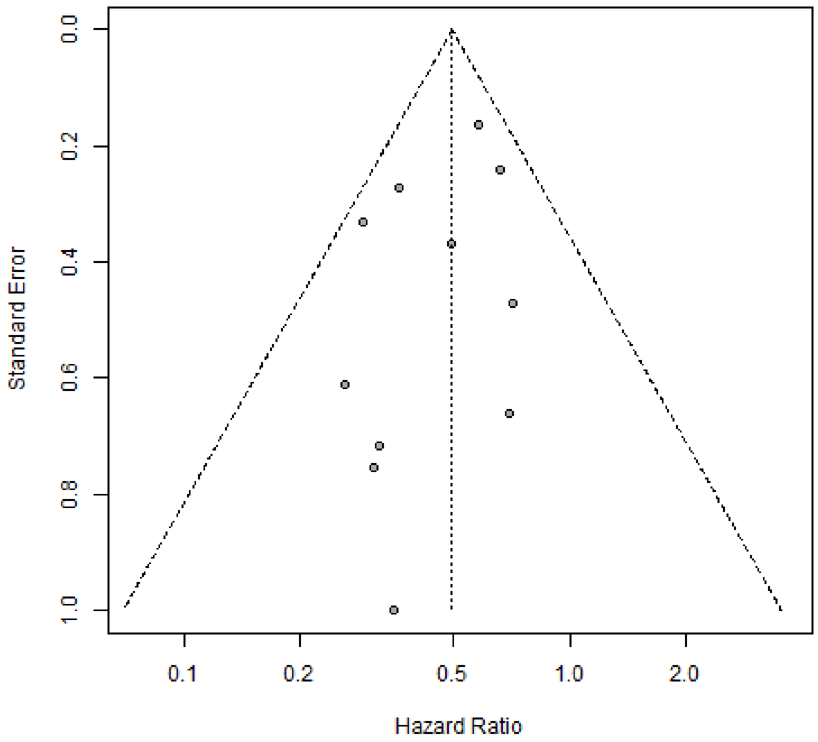

Supplementary Figure 1C: Funnel Plots of HR for OS by MPR Status (MPR vs no-MPR)

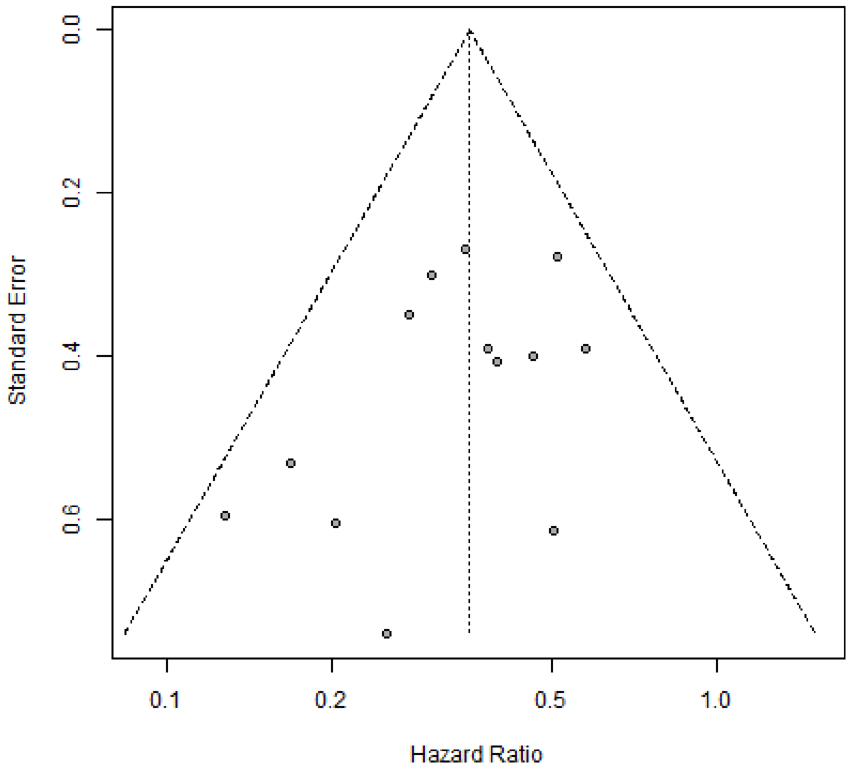

Supplementary Figure 1D: Funnel Plots of HR for EFS by MPR Status (MPR vs no-MPR)

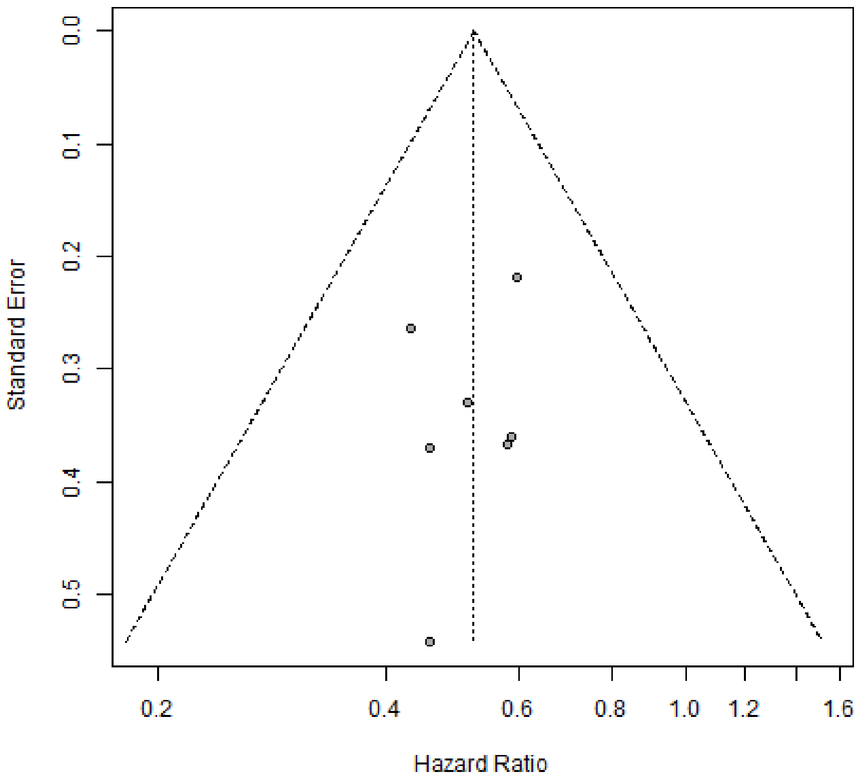

Supplementary Figure 2: Frequentist Analysis - Subgroup Analyses for OS by MPR Status (MPR vs no-MPR)

Subgroup	No. Studies	Sample Size	HR [95% CI]	Heterogeneity
**Overall**	**13**	**1193**	**0.36 [0.29,0.44]**	**0%**
**MPR definition**				
<=10% viable tumor cells	10	1041	0.39 [0.31,0.49]	0%
Less than 1/3	2	68	0.18 [0.08,0.40]	0%
Not defined	1	84	0.30 [0.17,0.55]	N/A
**Treatment group**				
CRT=>S	4	164	0.21 [0.12,0.37]	0%
CT/CRT=>S	2	139	0.33 [0.21,0.53]	0%
CT=>S	7	890	0.41 [0.31,0.53]	0%
**Trial design**				
RCT+other trial	3	105	0.20 [0.10,0.39]	0%
RWE	10	1088	0.38 [0.30,0.47]	0%
**Resection**				
Potentially resectable	1	84	0.30 [0.17,0.55]	N/A
Resected	12	1109	0.36 [0.29,0.46]	0%
**Race**				
Asian	3	152	0.25 [0.16,0.40]	0%
Non-Asian	10	1041	0.39 [0.31,0.49]	0%
**Taxane**				
Yes	3	105	0.20 [0.10,0.39]	0%
No	10	1088	0.38 [0.30,0.47]	0%
**Stage**				
Stage IIIB - none	4	247	0.30 [0.17,0.52]	49%
Stage IIIB - some	6	482	0.34 [0.25,0.48]	0%
Stage IIIB - unclear	3	464	0.41 [0.26,0.63]	7%
**Use of adjuvant chemotherapy**				
Adjuvant - all	1	37	0.25 [0.06,1.07]	N/A
Adjuvant - some	5	558	0.37 [0.27,0.50]	0%
Adjuvant - no	3	185	0.38 [0.19,0.75]	45%
Adjuvant - unclear	4	413	0.30 [0.20,0.46]	2%
**HR quality of estimate**				
Author reported HR	4	531	0.38 [0.28,0.53]	0%
HR reconstructed from KM, with n-at-risk	8	621	0.35 [0.26,0.48]	0%
HR reconstructed from KM, without n-at-risk	1	41	0.17 [0.06,0.48]	N/A
**Time zero on KMC**				
Surgery	8	763	0.33 [0.24,0.46]	10%
Tx Initiation	5	430	0.37 [0.28,0.50]	0%

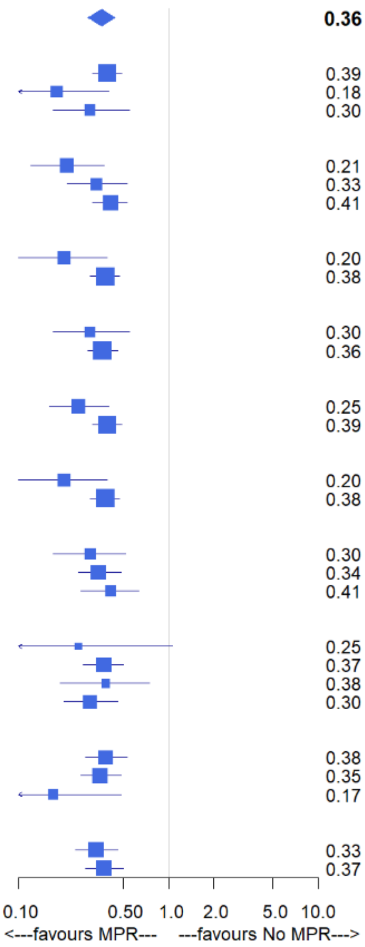

Notes: The size of the square represents the study's weight in the meta-analysis. The diamond represents the overall estimate. In the treatment subgroup, the number of studies does not add up to 12 but to 13. This is because Krantz et al. (2018) reported separately on subgroups CT=>S and CRT=>S. Thus the same study is counted in the 2 strata CT=>S and CRT=>S.

Legend: =>: followed by

Abbreviations: CI: Confidence interval; CRT: Chemoradiotherapy; CT: Chemotherapy; HR: Hazard ratio; KMC: Kaplan-Meier curve; MPR: Major pathological response; OS: Overall survival; RCT: Randomized controlled trial; RWE: Real-world evidence; S: Surgery; Tx: Treatment.

Supplementary Figure 3: Frequentist Analysis - HR for EFS by pCR Status (pCR vs no pCR)

Author (year)	Histology	Stage	Treatment	pCR	Sample Size	HR	95% CI	Weight
van der Meij BS 2011	All	III	CRT=>S	29%	51	0.26	[0.08; 0.86]	2.7%
Mouillet 2012*	All	IB−IIB	CT=>S	8%	492	0.29	[0.15; 0.56]	9.3%
Yamaguchi 2013*	All	IIIA/B	CRT=>S	23%	39	0.31	[0.07; 1.37]	1.8%
Coroller 2017	All	II−III	CRT=>S	15%	85	0.32	[0.08; 1.30]	2.0%
Brandt 2019*	All	IB−IIIA	CT=>S	4%	142	0.35	[0.05; 2.48]	1.0%
Kayawake 2019*	All	II−III	CT/CRT=>S	26%	145	0.36	[0.21; 0.61]	13.8%
Couñago 2019	All	IIIA	CT/CRT=>S	27%	117	0.50	[0.24; 1.02]	7.5%
Kim 2016*	All	I−IV	CRT=>S	12%	574	0.58	[0.42; 0.79]	37.7%
Lee H 2014	All	IIIA	CRT=>S	16%	355	0.66	[0.41; 1.06]	17.3%
Tanaka 2018*	All	IIIA	CRT=>S	29%	34	0.70	[0.19; 2.55]	2.3%
Remark 2016*	All	III	CT=>S	12%	122	0.71	[0.28; 1.78]	4.6%
**Random effects model**						**0.49**	**[0.41; 0.60]**	**100.0%**

Heterogeneity: $I^2 = 0\%$, $\tau^2 = 0$, $p = 0.53$

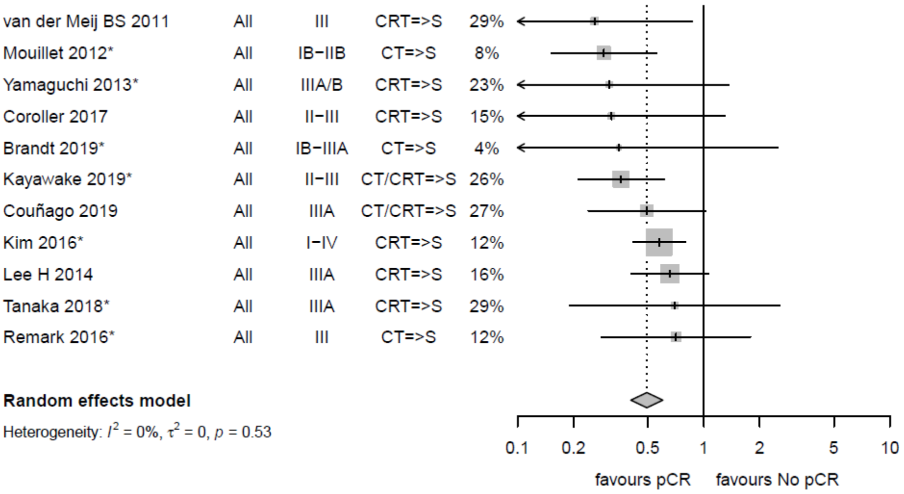

Note: an asterisk means that HR was reconstructed from Kaplan-Meier curves.

Legend: =>: followed by

Abbreviations: CI: Confidence interval; CRT: Chemoradiotherapy; CT: Chemotherapy; EFS: Event-free survival; HR: Hazard ratio; pCR: Pathologic complete response; S: Surgery

Supplementary Figure 4: Frequentist Analysis - Subgroup Analyses for EFS by pCR Status (pCR vs no-pCR)

Subgroup	No. Studies	Sample Size	HR [95% CI]	Heterogeneity
**Overall**	**11**	**2156**	**0.49 [0.41,0.60]**	**0%**
**pCR definition**				
0% viable tumor cells	3	648	0.46 [0.24,0.88]	35%
ypT0	2	929	0.60 [0.46,0.78]	0%
ypT0N0	3	298	0.44 [0.24,0.82]	0%
Not defined	3	281	0.34 [0.21,0.54]	0%
**Treatment group**				
CRT=>S	6	1138	0.56 [0.44,0.72]	0%
CT/CRT=>S	2	262	0.40 [0.26,0.62]	0%
CT=>S	3	756	0.40 [0.22,0.73]	17%
**Trial design**				
RCT+other trial	2	526	0.37 [0.17,0.81]	29%
RWE	9	1630	0.52 [0.42,0.64]	0%
**Resection**				
Potentially resectable	2	151	0.54 [0.29,1.01]	0%
Resected	9	2005	0.48 [0.38,0.60]	9%
**Race**				
Asian	5	1147	0.54 [0.43,0.68]	0%
Non-Asian	6	1009	0.39 [0.27,0.57]	0%
**Stage**				
Stage IIIB - none	5	1140	0.50 [0.35,0.70]	8%
Stage IIIB - some	6	1016	0.49 [0.38,0.63]	0%
**Use of adjuvant chemotherapy**				
Adjuvant - all	1	34	0.70 [0.19,2.55]	N/A
Adjuvant - some	7	1807	0.48 [0.05,2.48]	14%
Adjuvant - no	1	142	0.35 [0.38,0.61]	N/A
Adjuvant - unclear	2	173	0.46 [0.18,1.22]	41%
**HR quality of estimate**				
Author reported HR	4	608	0.54 [0.37,0.77]	0%
HR reconstructed from KM, with n-at-risk	7	1548	0.48 [0.37,0.61]	1%
**Time zero on KMC**				
Diagnosis	1	117	0.50 [0.24,1.02]	N/A
Surgery	6	1418	0.45 [0.33,0.62]	32%
Tx Initiation	4	621	0.56 [0.37,0.86]	0%

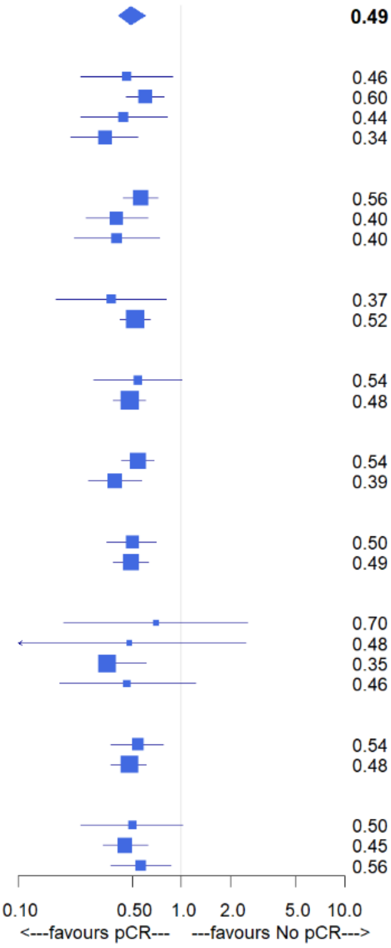

Notes: The size of the square represents the study's weight in the meta-analysis. The diamond represents the overall estimate. Taxane subgroup is not shown due to the fact that the taxane yes stratum was informed by only one study.

Legend: =>: followed by

Abbreviations: CI: Confidence interval; CRT: Chemoradiotherapy; CT: Chemotherapy; EFS: Event-free survival; HR: Hazard ratio; KMC: Kaplan-Meier curve; N/A: Not available; RCT: Randomized controlled trial; RWE: Real-world evidence; S: Surgery; Tx: Treatment; ypT0: absence of disease in the primary tumor (T) at surgery; ypT0N0: absence of disease in primary tumor (T) and lymph nodes (N) at surgery.

Supplementary Figure 5: Frequentist Analysis - HR for EFS by MPR Status (MPR vs no-MPR)

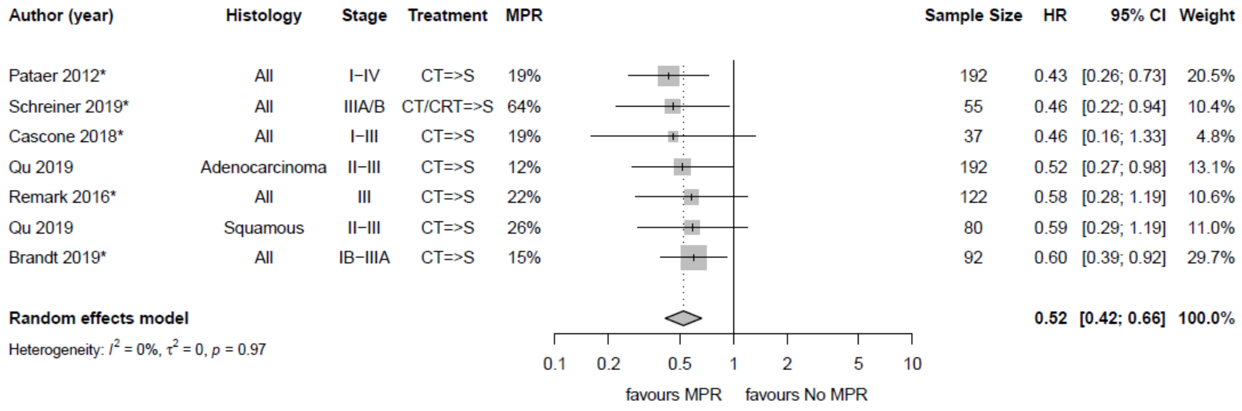

Note: an asterisk means that HR was reconstructed from Kaplan-Meier curves.

Legend: =>: followed by

Abbreviations: CI: Confidence interval; CRT: Chemoradiotherapy; CT: Chemotherapy; EFS: Event-free survival; HR: Hazard ratio; MPR: Major pathologic response; S: Surgery.

Supplementary Figure 6: Frequentist Analysis -Subgroup Analyses for EFS by MPR Status (MPR vs no-MPR)

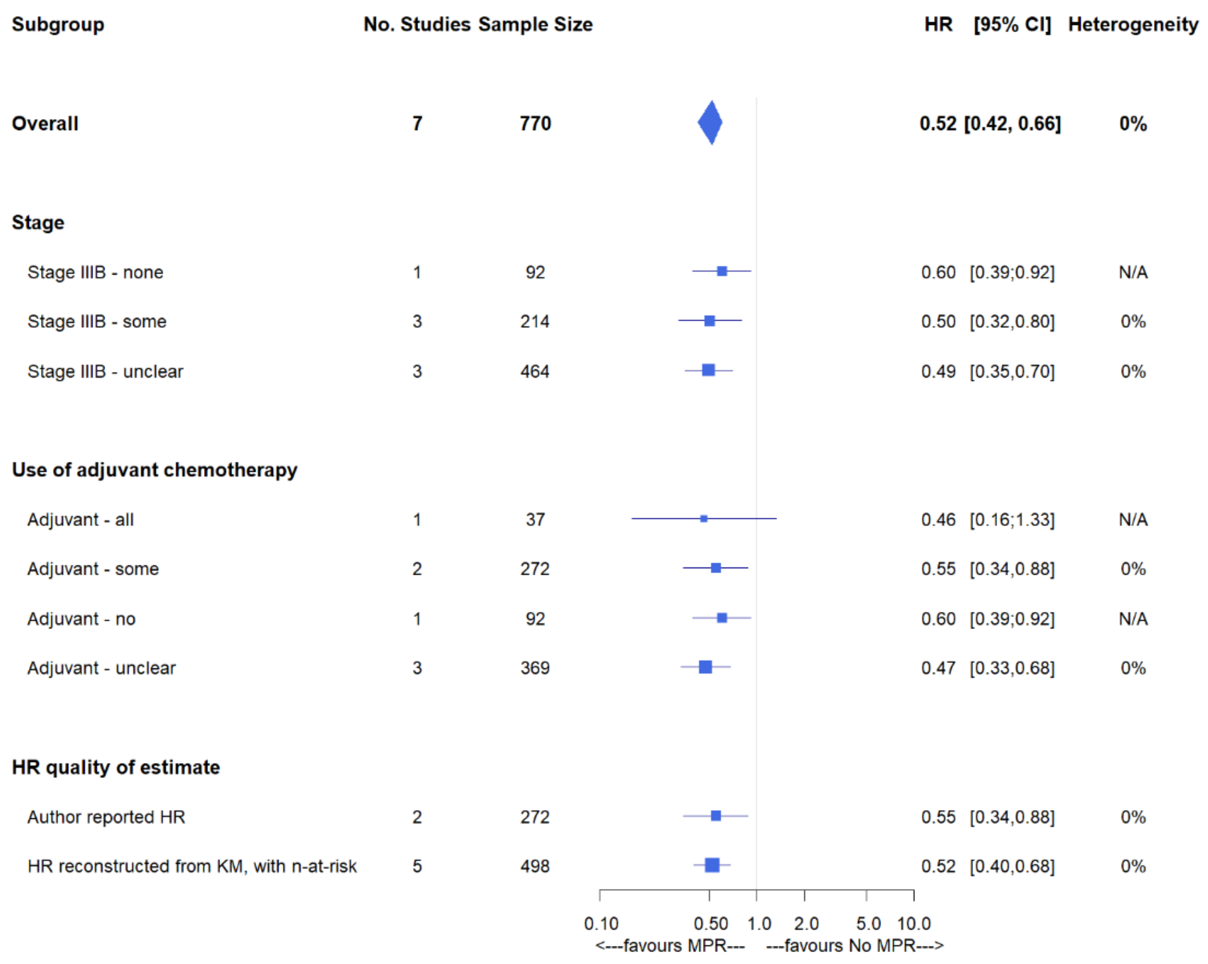

Notes: The size of the square represents the study's weight in the meta-analysis. The diamond represents the overall estimate. Many subgroups are not shown due to the fact that either all 7 studies informed only one stratum or 1 out of 2 strata was inform by only one study.

Abbreviations: CI: Confidence interval; EFS: Event-free survival; HR: Hazard ratio; KM: Kaplan-Meier; MPR: Major pathological response.

Supplementary Figure 7: Bayesian Analysis - Predicted RCT Treatment Effects on OS Given Treatment Benefits on pCR

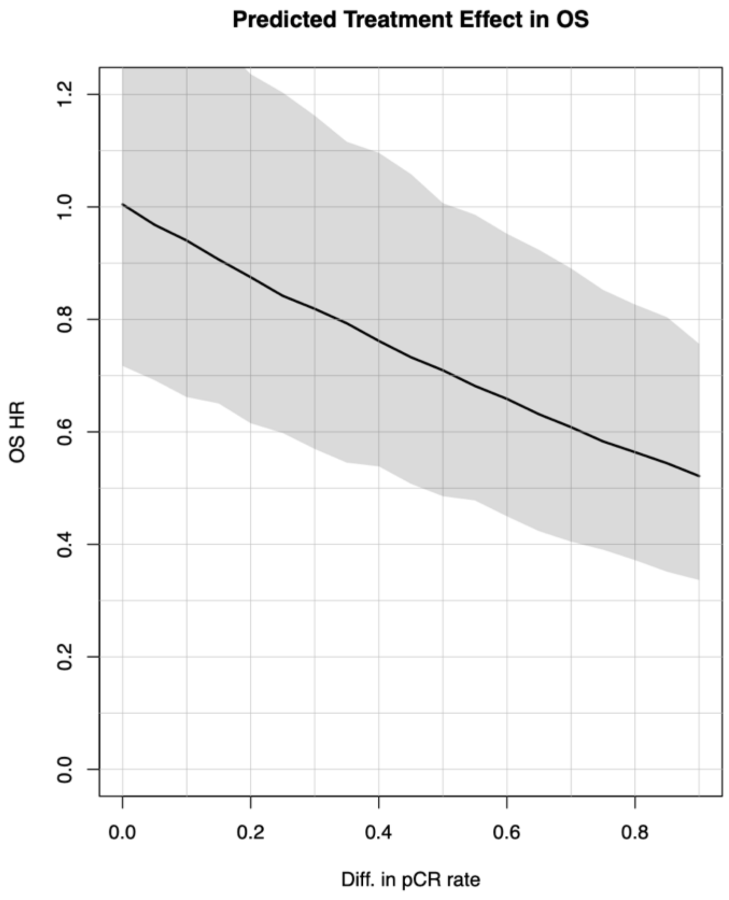

Treatment effect predictions of a future RCT are based on results of Bayesian analysis of OS vs. pCR and assume a 10% pCR rate on the control arm.

Solid line represents median predictions. Shaded grey region represents 95% probability interval for a trial with N=240 followed for a maximum of 6 years.

Abbreviations: HR: Hazard ratio; OS: Overall survival; pCR: Pathologic complete response; RCT: Randomized controlled trial

Supplementary Figure 8: Trial-level Analysis - HR for OS vs Odds Ratio for pCR

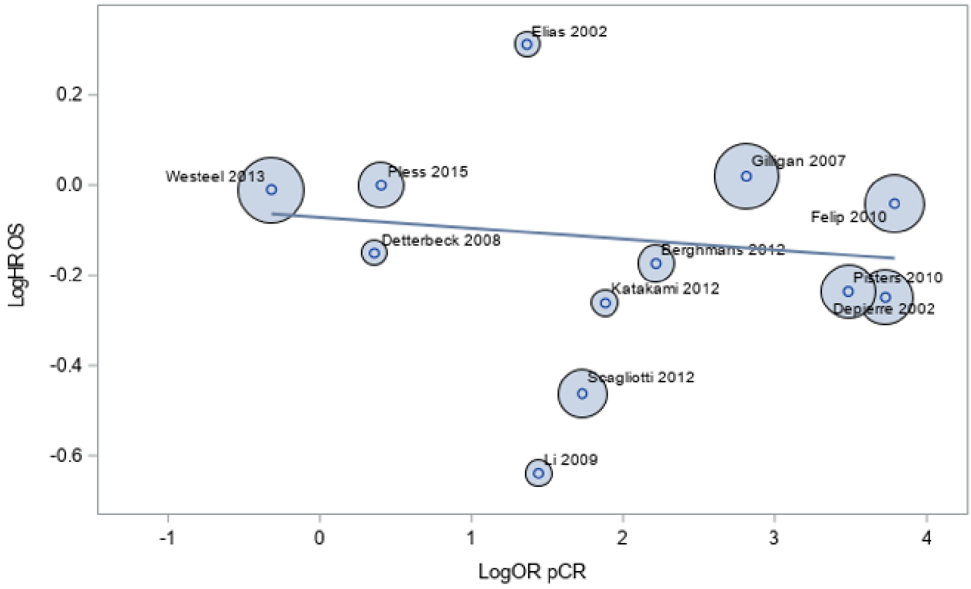

Parameters	Odds Ratio (OR) Model	Sensitivity Analysis
R^2 (95% CI), adjusted R^2	0.045 (-0.149, 0.239), -0.051	0.635 (0.147, 1.123), 0.453
Slope (SE; p-value)	-0.024 (0.035, 0.510)	-0.151 (0.081, 0.203)

Pearson's coefficient	Odds Ratio Model - Ln OR pCR	Sensitivity analysis – Ln OR pCR
$r_{Pearson}$ (95% CI)	-0.211 (-0.695, 0.420)	-0.797 (-0.994, 0.762))

Legend: The line is the linear regression; the circle represents the weight of each trial

Abbreviations: CI: Confidence interval; OR: Odds ratio; pCR: Pathologic complete response; RD: Risk difference; SE: Standard error

Supplementary Figure 9: Trial-level Analysis – HR for EFS vs Risk Difference for pCR

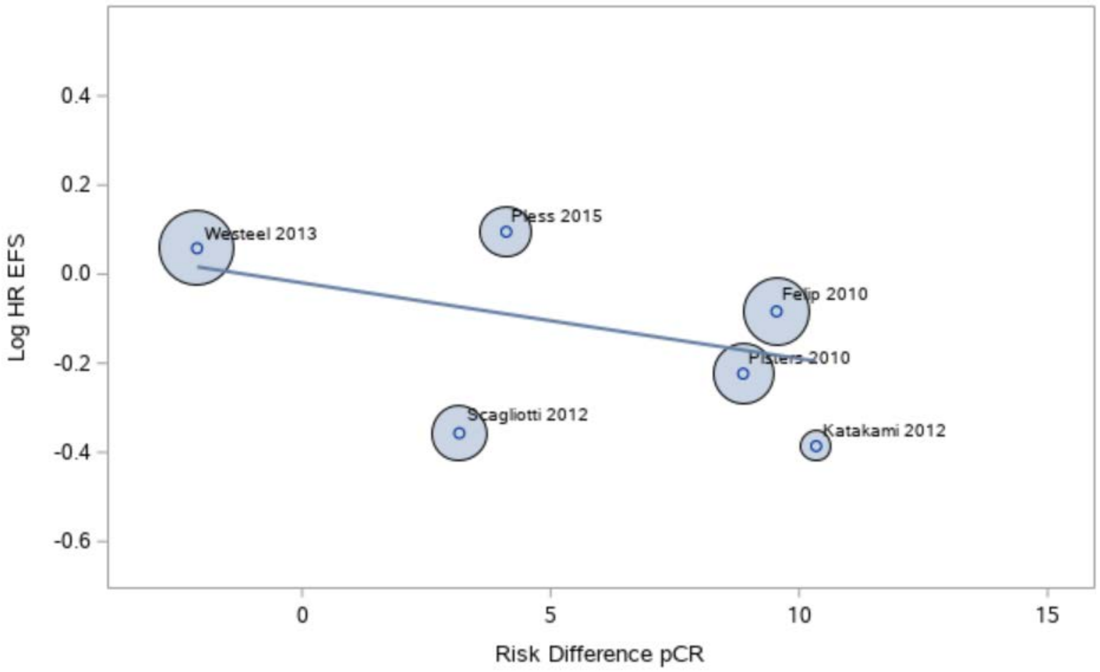

Parameters	Risk Difference Model for pCR	Sensitivity analysis
R^2 (95% CI), adjusted R^2	0.246 (-0.222, 0.714), 0.058	0.834 (0.277, 1.390), 0.668
Slope (SE; p-value)	-0.017 (0.015, 0.317)	-0.201 (0.090, 0.267)

Pearson's coefficient	Risk Difference Model for pCR	Sensitivity analysis
$r_{Pearson}$ (95% CI)	-0.496 (-0.926, 0.563)	-0.913 (N/A)

Legend: The line is the linear regression; a circle represents the weight of each trial

Abbreviations: CI: Confidence interval; EFS: event-free survival; HR: Hazard ratio; N/A: Not available; OR: Odds ratio; pCR: Pathological complete response; RD: Risk difference; SE: Standard error

Supplementary Figure 10: Trial-level Analysis - HR for EFS vs Odds Ratio for pCR

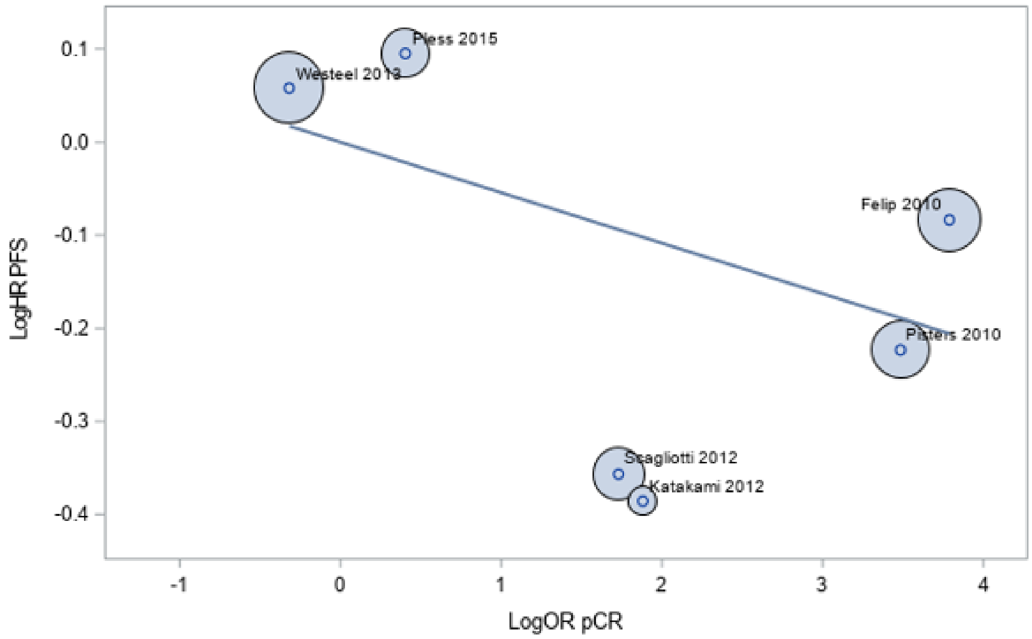

Parameters	Odds Ratio Model for pCR	Sensitivity analysis
R^2 (95% CI), adjusted R^2	0.319 (-0.162, 0.803), 0.149	0.834 (0.277, 1.390), 0.668
Slope (SE; p-value)	-0.0542 (0.040, 0.243)	-0.201 (0.090, 0.267)

Pearson's coefficient	Odds Ration Model for pCR	Sensitivity analysis
$r_{Pearson}$ (95% CI)	-0.565 (-0.937, 0.499)	-0.913 (N/A)

Legend: The line is the linear regression; the circle represents the weight of each trial

Abbreviations: CI: Confidence interval; OR: Odds ratio; pCR: Pathologic complete response; RD: Risk difference; SE: Standard error

Supplementary Figure 11: Correlation Between HR for OS and HR for EFS

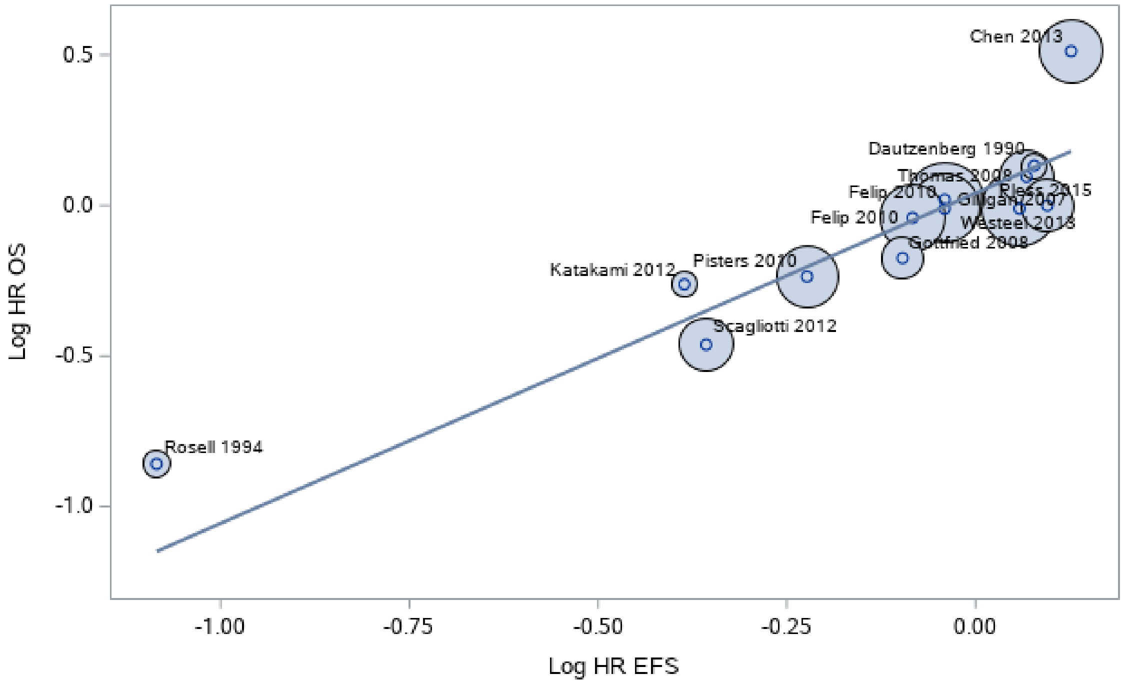

Variable	Result
Number of observations	13*
Parameters	2
Error DF	11
MSE	2.8696
R^2, adjusted R^2	0.7159, 0.6901
$r_{Pearson}$ (CI)	0.846 (0.528, 0.950)

Legend: The line is the regression line; the size of the circle represents the 95% confidence limit

*11 RCTs contributed 1 data point each. 1 RCT by Felip et al. (2010) contributed 2 data points.

Abbreviations: CI: Confidence interval; EFS: Event-free survival; HR: Hazard ratio; OS: Overall survival
